# *Codonopsis pilosula* Lipophilic Extract-Loaded Thermosensitive Nanogel Attenuates Skin Photoaging by Inhibiting the FGFR/PI3K/AKT/mTOR Pathway

**DOI:** 10.3390/pharmaceutics18070869

**Published:** 2026-07-16

**Authors:** Jiangtao Zhou, Yuhui Ge, Ran Li, Zhuoyang Cheng, Jianping Gao, Bin Zheng

**Affiliations:** 1 School of Pharmacy, Shanxi Medical University, Taiyuan 030001, China; zjt881206@sxmu.edu.cn (J.Z.);; 2 Medicinal Basic Research Innovation Center of Chronic Kidney Disease, Ministry of Education, Shanxi Medical University, Taiyuan 030001, China

**Keywords:** skin photoaging, *Codonopsis pilosula* lipophilic extract, thermosensitive nanogel, FGFR/PI3K/AKT/mTOR signaling, molecular docking

## Abstract

**Background:** Skin photoaging, primarily induced by chronic ultraviolet (UV) radiation exposure, is characterized by dryness, wrinkle formation, pigmentation abnormalities, and reduced skin elasticity, resulting from oxidative stress, inflammation, and degradation of the extracellular matrix. *Codonopsis pilosula*, a traditional food–medicine homologous plant, is recognized for its anti-aging properties. However, its lipophilic components (designated as CP-L) remain insufficiently explored. **Methods:** Herein, we developed a thermosensitive nanogel encapsulating CP-L-loaded transferosomes (CP-L nanogel) to enhance topical delivery and evaluated its effects in both a UV-induced photoaging mouse model and UVB-irradiated HaCaT keratinocytes. **Results:** In UV-induced mice, topical application of the nanogel markedly reduced skin wrinkling and epidermal hyperplasia, with epidermal thickness decreased by 83.2% compared to the model group (*p* < 0.01), and restored skin elasticity and collagen deposition, as evidenced by a 35.5% increase in collagen area fraction (*p* < 0.01). Correspondingly, in UVB-irradiated HaCaT cells, it significantly increased cell viability from 53.0 ± 9.6% to 89.4 ± 1.0% (*p* < 0.01) and suppressed apoptosis from 30.1 ± 0.48% to 12.4 ± 0.66% (*p* < 0.01). Furthermore, the CP-L nanogel consistently attenuated oxidative stress, with SOD, CAT, and GSH-Px activities increased by 73.1%, 188.1%, and 18.2%, respectively (*p* < 0.01), and MDA levels reduced by 71.0% (*p* < 0.01), while inflammatory responses were suppressed, as TNF-α, IL-1α, IL-1β, and IL-6 levels decreased by 28.3%, 22.6%, 12.8% and 31.9%, respectively (*p* < 0.01). Mechanistically, transcriptomic and molecular analyses revealed that the nanogel potently inhibited the UV-induced activation of the FGFR/PI3K/AKT/mTOR/p70S6K signaling cascade at both transcriptional and protein levels, with the phosphorylation levels of FGFR, PI3K, AKT, mTOR, and p70S6K significantly reduced by 43.9%, 30.9%, 38.8%, 34.9%, and 57.3%, respectively (*p* < 0.01). Molecular docking and dynamics simulations identified isofuranodienone and aromadendrene oxide-(2) as key constituents with high-affinity, stable binding to FGFR1 and AKT1. The cytoprotective effect of the nanogel was completely abolished by co-treatment with the FGFR inhibitor PD173074, confirming functional reliance on this pathway. Enhanced cellular delivery of the formulation was directly demonstrated by flow cytometry, showing an approximately 1.8-fold increase in cellular uptake compared to the free drug (*p* < 0.01). **Conclusions:** Collectively, these results demonstrated that the CP-L nanogel alleviated skin photoaging through a multi-faceted mechanism involving enhanced cellular delivery, potent antioxidant and anti-inflammatory activities, and specific inhibition of the FGFR/PI3K/AKT/mTOR signaling cascade, highlighting its potential as a multitargeted topical agent derived from an edible plant.

## 1. Introduction

Skin photoaging is primarily induced by UV radiation and clinically manifests as dryness, coarse wrinkles, irregular pigmentation, and reduced elasticity [1,2]. Its pathogenesis involves a self-perpetuating cycle initiated by chronic UV exposure, which induces mitochondrial DNA mutations and excessive ROS generation [3], ultimately leading to impairment of mitochondrial membrane potential and respiratory function. This mitochondrial dysfunction can trigger apoptosis, mitophagy, and cellular senescence, thereby accelerating photodamage and photoaging [4,5]. Furthermore, photoaging is characterized by an imbalance between the synthesis and degradation of extracellular matrix (ECM) components [6], a process largely driven by the upregulated activity of matrix metalloproteinases (MMPs) [7]. Both intrinsic and extrinsic stimuli can activate specific MMPs [8] through complex mechanisms involving oxidative stress, collagen degradation, and dysregulation of multiple signaling pathways [9]. Given this multifactorial pathogenesis, therapeutic agents capable of multi-target intervention are critically needed. Botanical extracts, with their complex mixtures of bioactive compounds, are particularly well-suited to address these interconnected pathways. Accordingly, the development of safe and effective anti-photoaging agents from medicinal plants has emerged as a significant focus in dermatological research.

*Codonopsis pilosula* is widely used in traditional medicine and as a dietary component. It has long been valued for its health-promoting properties and is regarded as a source of essential nutrients as well as a functional food with wellness-supporting potential. This dual role as a food–medicine homologous material underscores its importance in both nutritional and health-related applications [10]. Previous studies have reported multiple bioactivities of *Codonopsis*, including immunomodulatory, gastroprotective, and anti-aging effects [11,12]. Notably, lipophilic components derived from various edible and medicinal plants have demonstrated promising efficacy in alleviating UV-induced skin damage [13,14,15,16,17,18], supporting the potential application of plant-derived lipophilic fractions in skin health. Among these medicinal plants, *Codonopsis pilosula* stands out as a particularly compelling candidate. In traditional Chinese medicine, the root of *Codonopsis pilosula* has been historically documented as a tonic agent for “supplementing vital energy (Qi)” and “nourishing blood,” with its decoctions being prescribed for debility, fatigue, and age-related decline [19,20]. Notably, ancient herbal classics also mention its use for “moistening the skin” and “delaying senility,” providing ethnopharmacological clues to its potential anti-photoaging properties. However, while modern phytochemical investigations have extensively characterized its water-soluble polysaccharides, the volatile oils and other lipophilic constituents—which are more likely to penetrate the skin barrier—have not yet been examined for their photoprotective potential. This gap, combined with the traditional skin-benefiting claims, prompted us to prioritize the lipophilic fraction of *Codonopsis pilosula* for this topical delivery study. Despite these promising clues, however, *Codonopsis* contains diverse bioactive constituents, including polysaccharides, terpenoids, and volatile oils [21], its lipophilic components (designated as CP-L) remain relatively underexplored. Based on the established roles of botanical lipophilic extracts in maintaining skin barrier integrity, mitigating oxidative stress, and modulating inflammatory responses, it is hypothesized that the CP-L may serve as a functional ingredient for the prevention of photoaging, potentially through the regulation of specific signaling pathways.

Nevertheless, the therapeutic application of CP-L faces two major challenges: its inherent poor skin permeability and chemical instability, which severely limit topical bioavailability and efficacy. To overcome these limitations, we designed an advanced delivery strategy utilizing transferosomes (TFs). TFs have attracted considerable attention for transdermal delivery due to their cellular affinity, low immunogenicity, and favorable biocompatibility [22]. As a specialized class of liposomes, TFs are ultra-deformable vesicles composed of phospholipids and edge-activating surfactants [23]. Their bilayer structure, which closely mimics biological membranes, confers excellent biocompatibility and membrane fusion potential [24]. The surfactants impart high deformability, enabling TFs to effectively penetrate the skin barrier and enhance the delivery of encapsulated compounds [25], directly addressing the permeation issue of CP-L. Despite these advantages, liquid-state TFs are susceptible to drug leakage and rapid clearance [26]. To address this, we incorporated the CP-L-loaded TFs into a thermosensitive hydrogel. This integrated design not only stabilizes the vesicles but also prolongs cutaneous residence time, thereby enhancing drug exposure and therapeutic efficacy [27], offering a comprehensive solution to the stability and retention challenges. Unlike conventional hydrogels, the thermosensitive formulation allows uniform dispersion of TFs. Its temperature-responsive sol-gel transition at skin temperature facilitates easy application and enables sustained drug release [28,29].

On the molecular level, accumulating evidence highlights the pivotal role of the FGFR and its downstream PI3K–AKT–mTOR–p70S6K signaling axis in maintaining skin homeostasis [30,31]. This pathway plays a critical role in regulating fibroblast proliferation, cellular survival under oxidative stress, and the synthesis of key ECM components, particularly type I collagen [32]. However, chronic UV exposure markedly disrupts this signaling cascade, resulting in reduced collagen production and accelerated ECM degradation. Therefore, targeted modulation of the FGFR–PI3K–AKT–mTOR–p70S6K pathway has emerged as a promising therapeutic strategy for photoaging. Despite this promise, it remains unknown whether the lipophilic extract of *Codonopsis pilosula* exerts its protective effects by influencing this specific pathway.

In this study, we developed a thermosensitive nanogel loaded with CP-L-encapsulated transferosomes (CP-L nanogel) to enhance the stability, cutaneous delivery, and efficacy of the active lipophilic components. Its protective effects were systematically evaluated using a UV-induced photoaging mouse model and UVB-irradiated human HaCaT keratinocytes. Moving beyond phenotypic and histological assessments, our investigation comprehensively examined the nanogel’s antioxidant and anti-inflammatory capacities, its regulation of apoptosis, and—most critically—its modulation of the FGFR/PI3K-AKT/mTOR pathway at transcriptional and protein levels. To decipher the underlying mechanism, molecular docking and dynamics simulations were employed to predict the interaction of key CP-L constituents with the target proteins FGFR1 and AKT1. The functional dependence of the CP-L nanogel on the FGFR pathway was further verified using a specific inhibitor. Additionally, the enhanced cellular delivery efficiency of the nanogel system was quantitatively evaluated. This multi-faceted approach was designed to provide deep mechanistic insights into the anti-photoaging activity of the CP-L nanogel and to robustly support its potential as a safe, effective, and multitargeted topical agent derived from a food–medicine homologous plant.

## 2. Materials and Methods

### 2.1. Plant Material

The dried root of *Codonopsis pilosula* (Franch.) Nannf. was purchased from Zhen Dong Chinese Herbal Medicine Company and authenticated by Professor Jianping Gao (Shanxi Medical University, Shanxi, China). A voucher specimen (specimen No. CP-2026-001) has been deposited in the Herbarium of Shanxi Medical University.

### 2.2. Extraction of CP-L Components and Characterization by GC-TOF-MS

The lipophilic extract of *Codonopsis pilosula* (CP-L) was obtained as a pale yellow, oily substance. Dried root material (100 g) was ground into a coarse powder and extracted twice with 800 mL of 95% ethanol under reflux (80 °C, 2 h each). The combined ethanol extracts were concentrated under reduced pressure to yield a crude extract (yield: 18.5 ± 1.2 g). This crude extract was then suspended in distilled water (200 mL) and successively partitioned three times with an equal volume of petroleum ether (60–90 °C). The petroleum ether phases were combined, dried over anhydrous sodium sulfate, and evaporated under reduced pressure to obtain the lipophilic fraction (CP-L). The final extraction yield of CP-L was 4.2 ± 0.3 g per 100 g of dried raw material (4.2%, *w*/*w*).

The chemical composition of CP-L was analyzed by gas chromatography–time-of-flight mass spectrometry (GC-TOF-MS). Analysis was performed on an Agilent 7890B GC system coupled to a TOF mass spectrometer and equipped with an Agilent DB-WAX capillary column (30 m × 0.25 mm × 0.25 µm). Helium was used as the carrier gas at a constant flow rate of 1.0 mL/min. A 1 µL sample was injected in splitless mode with the injector temperature set at 245 °C. The GC oven temperature was programmed as follows: 40 °C (held for 3 min), increased to 105 °C at 6 °C/min, then to 180 °C at 4 °C/min, and finally to 245 °C at 10 °C/min (held for 5 min). The ion source temperature was maintained at 220 °C. The mass spectrometer operated in full-scan mode (*m*/*z* 35–450) with an ionization voltage of 70 eV and a scan rate of 15 spectra per second (15 Hz). Mass spectra were matched against the NIST 17 (National Institute of Standards and Technology, version 2.3) mass spectral library. For data processing, peaks were identified by matching their mass spectra with the NIST 17 library, and only those with a match quality >80% were considered for positive identification. Compounds with a relative content below 0.1% were excluded due to their minor contribution to the overall profile. After excluding solvent-related and contaminant peaks, the 20 compounds with the highest relative contents, collectively accounting for the vast majority of the total peak area of the identified natural product profile.

### 2.3. Preparation and Characterization of Transferosomes

#### 2.3.1. Preparation of Transferosomes

CP-L-loaded transferosomes (CP-L-TFs) were prepared using the ethanol injection method. The organic phase consisted of 1 mL of anhydrous ethanol containing egg yolk lecithin (EPC, 20 mg) and CP-L (4 mg). The aqueous phase comprised distilled water (4 mL) containing sodium deoxycholate (NaDC, 7 mg). The organic phase was added dropwise to the aqueous phase under magnetic stirring at 1000 rpm and 40 °C. The mixture was continuously stirred at 40 °C for 1 h, followed by solvent removal under reduced pressure to eliminate residual ethanol.

The particle size, polydispersity index (PDI), and zeta potential of CP-L-TFs were determined using a dynamic light scattering analyzer (Zetasizer Nano ZS, Malvern, UK). The morphological characteristics of CP-L-TFs were examined using transmission electron microscopy (TEM; JEM-1200EX, JEOL, Tokyo, Japan). Due to the poor water solubility of CP-L, a low-speed centrifugation method (10,000 rpm, 10 min) was employed to separate non-entrapped (free) CP-L from CP-L-TFs. After centrifugation, 100 μL of the supernatant, which contained the CP-L-TFs, was carefully collected and diluted with 900 μL of methanol to disrupt the vesicular bilayer structure and fully release the encapsulated CP-L. The concentration of CP-L in the supernatant was then determined using a UV–visible spectrophotometer (UV-1200, NAPADA, Shanghai, China) at a detection wavelength of 227 nm. This wavelength was selected based on a preliminary UV–Vis absorption scan of CP-L in methanol, which revealed a characteristic maximum absorbance peak at 227 nm. The entrapment efficiency (EE) and drug loading (DL) of CP-L-TFs were calculated using Equations (1) and (2), respectively:

EE (%) = m_entrap_/m_total_ × 100 (1)

where m_entrap_ represents the mass of CP-L encapsulated within CP-L-TFs, as quantified from the supernatant after methanol disruption, and m_total_ denotes the total mass of CP-L initially added during preparation.

DL (%) = m_entrap_/m_all_ × 100 (2)

where m_entrap_ represents the mass of CP-L encapsulated within CP-L-TFs, as quantified from the supernatant after methanol disruption, and m_all_ denotes the total mass of encapsulated CP-L, EPC, and NaDC.

The storage stability of CP-L-TFs was evaluated by measuring particle size and EE for up to 7 days at 4 °C.

#### 2.3.2. Preparation and Characterization of the Thermosensitive Nanogel (CP-L Nanogel)

The thermosensitive nanogel encapsulating CP-L-TFs (denoted as CP-L nanogel) was prepared using the cold method. Briefly, Pluronic F127 (0.86 g) and Pluronic F68 (0.14 g) were dissolved in the CP-L-TFs suspension (3.00 g) under magnetic stirring (1000 rpm) in an ice-water bath. The mixture was kept at 4 °C for 12 h to obtain a homogeneous 25% (*w*/*w*) gel precursor solution. Gelation was induced by warming the precursor solution to 32 °C. A blank hydrogel was prepared identically but using distilled water instead of the CP-L-TFs suspension.

For physicochemical characterization, samples of CP-L-TFs, blank hydrogel, and CP-L nanogel were lyophilized (FD-12A-50T, SHUNZHI, Shanghai, China). Potential interactions between components were analyzed by X-ray diffractometer (Smartlab, Rigaku, Tokyo, Japan) and Fourier-transform infrared (FT-IR) spectroscopy (Nicolet iS5, Thermo Fisher Scientific, Waltham, MA, USA). The XRD analysis was performed by scanning at a speed of 10° min^−1^ in the 2*θ* range of 5–80°. The FT-IR analysis was performed by scanning from 400 to 4000 cm^−1^ at a spectral resolution of 4 cm^−1^. The thermosensitive sol-gel transition was evaluated by the tube inversion method. Injectability was assessed by expelling the precursor solution from a syringe into distilled water at 32 °C. The microstructure of the freeze-dried gels was examined by scanning electron microscopy (SEM; SU 8020, Hitachi, Tokyo, Japan) after sputter-coating with gold, using an accelerating voltage of 10 kV.

The hemolysis test was conducted to evaluate the blood compatibility of CP-L nanogel. A range of concentrations (0–80 μg/mL) of CP-L nanogel were mixed with 2% red blood cell suspension, and the PBS (pH 7.4) and deionized water were used as negative and positive controls. After incubating at 37 °C for 3 h, the supernatant was separated by centrifugation (2300 rpm, 15 min), and the absorbance was measured at 540 nm using a microplate reader; the hemolysis rate was calculated.

### 2.4. Animal Model Establishment and Grouping

Thirty-six female specific pathogen-free (SPF) KM mice (6 weeks old, 25 ± 2 g; Approval No. SYXK(Jing)2019-0007) were provided by Beijing Speifu Biotechnology Co., Ltd. (Beijing, China). Following a 7-day acclimation period with free access to water, all animals were housed under controlled SPF conditions at a temperature of 22 ± 2 °C, relative humidity of 50 ± 1%, and a 12 h light/dark cycle. Environmental parameters, including air quality, noise levels, and cleanliness, were strictly monitored. All experimental procedures were approved by the Animal Care and Use Committee of Shanxi Medical University and were conducted in accordance with AAALAC and IACUC guidelines. Euthanasia was performed by cervical dislocation.

The mice were randomly assigned to six groups (*n* = 6 per group): (1) Control group, which received no UV irradiation; (2) Model group, which was exposed to UV irradiation without any topical treatment; (3) Vehicle group, which was exposed to UV irradiation and topically administered blank thermosensitive hydrogel without CP-L; (4) CP-L group, which was exposed to UV irradiation and topically administered unformulated CP-L; (5) CP-L nanogel group, which was exposed to UV irradiation and topically administered the developed CP-L-TFs thermosensitive nanogel; and (6) Positive group (Vitamin E, VE), which was exposed to UV irradiation and topically administered VE. Except for the Control group, all groups were subjected to chronic combined UVA and UVB irradiation for 8 weeks to establish a photoaging model. During this period, each treatment group received topical application of the corresponding formulation at a uniform dose of 3 mg. The dosage of CP-L was determined based on previous literature and preliminary experiments [33].

The UV irradiation protocol was conducted five days per week for eight consecutive weeks. A bank of TL-20W/12 RS UVB lamps (Philips, Amsterdam, The Netherlands) emitting a spectrum of 280–320 nm was used as the UV source. One minimal erythema dose (MED) was defined as 702 mJ/cm^2^. During the first week, mice were irradiated for 20 min per session, corresponding to 1 MED. The irradiation duration was increased by 20 min each subsequent week, corresponding to an additional 1 MED per week, until a maximum exposure time of 80 min, equivalent to 4 MED, was reached by the end of the experiment. Throughout the experimental period, dorsal skin appearance was photographed, and skin condition scores and thickness measurements were recorded. At the end of the eighth week, mice were euthanized by cervical dislocation. Blood samples were collected from the orbital sinus, and serum was separated by centrifugation at 1000× *g* for 10 min at 4 °C. Dorsal skin tissues were either fixed in 4% paraformaldehyde for histological analyses or stored at −80 °C for subsequent biochemical analyses.

### 2.5. Histological Analysis

#### 2.5.1. Hematoxylin and Eosin (H&E) Staining

Paraffin-embedded skin tissues were sectioned and stained with H&E. The stained sections were examined under a light microscope to evaluate epidermal thickness and structural alterations in the dermis.

#### 2.5.2. Masson’s Trichrome Staining

Collagen fiber distribution in skin tissues was visualized using Masson’s trichrome staining, in which collagen fibers appear blue. The stained areas were quantitatively analyzed using ImageJ software (version 1.54r).

#### 2.5.3. Elastin Van Gieson (EVG) Staining

Elastic fibers were visualized using EVG staining, in which elastic fibers are stained black. The relative content of elastic fibers in the dermis was quantified using ImageJ software.

#### 2.5.4. Immunohistochemistry (IHC)

IHC was performed on paraffin-embedded skin sections to assess the expression of MMP-1, LC3B, and collagen I. Following deparaffinization and rehydration, antigen retrieval was carried out by heating sections in citrate buffer. Endogenous peroxidase activity was blocked with 3% H_2_O_2_. Sections were then blocked and incubated sequentially with specific primary antibodies and corresponding secondary antibodies. Immunoreactivity was visualized using a DAB substrate, and nuclei were counterstained with hematoxylin. After dehydration through a graded ethanol series and clearing in xylene, sections were mounted. Images were captured and quantitatively analyzed using Image-Pro Plus software (version 7.0). Data processing and graph generation were performed using GraphPad Prism software (version 9.5).

### 2.6. Cell Culture

The human immortalized keratinocyte cell line HaCaT (Cat. No. TCH-C388) was purchased from Fujian Haixing Biological Co., Ltd. (Fuzhou, China) and cultured in Dulbecco’s Modified Eagle Medium (DMEM, Gibco, Waltham, MA, USA, Cat. No. 11965-092) supplemented with 10% fetal bovine serum (FBS, ExCell Bio, Shanghai, China, Cat. No. FSP500) and 1% penicillin/streptomycin (Solarbio, Beijing, China, Cat. No. P1400) at 37 °C in a humidified atmosphere containing 5% CO_2_. Cells in the logarithmic growth phase were harvested by trypsinization, counted and seeded at appropriate densities in different culture plates for subsequent experiments.

### 2.7. UVB Irradiation for Photoaging Model Induction and Cell Viability Assay (CCK-8)

Cell viability was assessed using the CCK-8 assay. HaCaT cells were seeded into 96-well plates at a density of 5 × 10^4^ cells/mL. After a 24 h incubation to allow cell attachment, the culture medium was aspirated, and the cell monolayer was gently washed twice with pre-warmed PBS to remove serum residues. A thin, uniform layer of PBS was then added to cover the cells. Cells were exposed to a single dose of UVB radiation (wavelength: 280–320 nm) using a calibrated UVB lamp (Philips, TL-20W). Based on preliminary experiments and the cited literature [34], an optimal dose of 240 mJ/cm^2^ was applied to induce photoaging damage in HaCaT cells. To evaluate the cytotoxic effects of CP-L and CP-L nanogel, a separate dose–response experiment was first performed. Cells were treated with a range of concentrations (0–80 μg/mL) of either unformulated CP-L or CP-L nanogel for 24 h, and cell viability was measured as described below. For the protective-effect study, cells were randomly assigned to six experimental groups: (1) Control group: no UVB irradiation, no treatment; (2) Model group: UVB irradiation only; (3) Vehicle group: UVB irradiation + blank thermosensitive hydrogel (without CP-L); (4) CP-L group: UVB irradiation + unformulated CP-L; (5) CP-L nanogel group: UVB irradiation + CP-L-TFs thermosensitive nanogel; (6) Positive group: UVB irradiation + VE. The final concentrations of CP-L used in the CP-L and CP-L nanogel groups were selected based on the preceding dose–response experiment. Immediately after UVB exposure, PBS was replaced with 100 µL of fresh complete medium containing the respective treatments. Following 24 h of incubation, 10 µL of CCK-8 reagent was added to each well. The plate was further incubated in the dark at 37 °C for 1–2 h, and the absorbance was measured at 450 nm using a microplate reader (Allsheng AMR-100, Hangzhou, China). Wells with medium and CCK-8 reagent but no cells served as blanks. Cell viability was calculated as a percentage relative to the Control group.

### 2.8. Detection of Apoptosis by Annexin V-FITC/PI Double Staining and Flow Cytometry

Apoptosis in HaCaT cells following UVB irradiation was analyzed using Annexin V-fluorescein isothiocyanate (FITC) and propidium iodide (PI) double staining followed by flow cytometry. After 24 h of treatment according to the aforementioned six experimental groups (Control, Model, Vehicle, CP-L, CP-L nanogel, and Positive group), cells were harvested by gentle trypsinization (using 0.25% trypsin without EDTA). The cell suspension was then centrifuged at 300× *g* for 5 min, and the pellet was washed twice with ice-cold PBS. Subsequently, cells were resuspended in 100 µL of 1× Annexin V Binding Buffer at a density of approximately 1 × 10^6^ cells/mL. Following the manufacturer’s protocol (Annexin V-FITC Apoptosis Detection Kit, Dalian Meilun Biotechnology Co., Ltd., Dalian, China, Cat. No. MA0220), 5 µL of Annexin V-FITC and 5 µL of PI staining solution were added to the cell suspension. After gentle mixing, cells were incubated at room temperature in the dark for 15 min. Then, 400 µL of 1× Binding Buffer was added, and samples were analyzed within 1 h using a flow cytometer (CytoFLEX, Beckman Coulter, Brea, CA, USA). For each sample, at least 10,000 events were acquired. Data analysis was performed using FlowJo software (version 14.0). Cells were classified into four distinct populations based on fluorescence signals: viable cells (Annexin V^−^/PI^−^), early apoptotic cells (Annexin V^+^/PI^−^), late apoptotic (Annexin V^+^/PI^+^), and necrotic/damaged cells (Annexin V^−^/PI^+^). The percentage of total apoptotic cells (early + late apoptotic) was calculated for statistical comparison among groups. The experiment was performed in triplicate.

### 2.9. Measurement of Intracellular ROS Levels

HaCaT cells were seeded in 6-well plates at a density of 1.5 × 10^5^ cells per well and cultured overnight. Following UVB irradiation, the cells were treated accordingly for 6 h. Subsequently, cells were incubated at 37 °C for 40 min with the fluorescent probe 2′,7′-dichlorodihydrofluorescein diacetate (DCFH-DA, Sigma-Aldrich, St. Louis, MO, USA, Cat. No. D6883) at a final concentration of 10 µM. After washing with PBS, the intracellular ROS levels were immediately detected using a flow cytometer (CytoFLEX, Beckman Coulter, USA) and analyzed with FlowJo software (version 14.0). The mean fluorescence intensity (MFI) was proportional to the intracellular ROS level.

### 2.10. Biochemical Assays

#### 2.10.1. Measurement of Oxidative Stress Markers

Levels of oxidative stress markers in skin tissue homogenates and HaCaT cell lysates, including superoxide dismutase (SOD; Cat. No. BC5165), malondialdehyde (MDA; Cat. No. BC0025), glutathione peroxidase (GSH-Px; Cat. No. BC1175), and catalase (CAT; Cat. No. BC0205), were measured using commercially available assay kits according to the manufacturers’ instructions (Beijing Solabio Technology Co., Ltd., Beijing, China).

#### 2.10.2. Measurement of Inflammatory Cytokines

The concentrations of inflammatory cytokines, including IL-1α (Cat. No. MM-0168M1), IL-1β (Cat. No. MM-0040M1), IL-6 (Cat. No. MM-0163M1), and TNF-α (Cat. No. MM-0132M1), MMP-1 (Cat. No. MM-61512R2), Collagen I (Cat. No. MM-0706M1) in skin tissue samples and HaCaT cell lysates were determined using enzyme-linked immunosorbent assay kits (Jiangsu Enzyme Immunoassay Industry Co., Ltd., Nanjing, China).

### 2.11. Transcriptome Sequencing and Library Preparation

Following quality assessment, eukaryotic mRNA was enriched using Oligo(dT)-coupled magnetic beads and subsequently fragmented in fragmentation buffer. First-strand complementary DNA (cDNA) was synthesized using random hexamer primers, followed by second-strand cDNA synthesis through the addition of buffer, dNTPs, and DNA polymerase I. The resulting double-stranded cDNA was purified using AMPure XP beads, subjected to end repair, A-tailing, and adapter ligation, and then size-selected using AMPure XP beads. The libraries were amplified by PCR to generate final sequencing libraries.

Transcriptome sequencing was performed on the Illumina/BGI platform. Raw sequencing data in Fastq format were processed using in-house Perl scripts to remove adapter sequences and low-quality reads containing more than 5% N bases or low-quality scores. Clean reads were aligned to the reference genome using HISAT2 software (version 2.2.2). Gene expression levels were quantified as fragments per kilobase of transcript per million mapped reads. Differentially expressed genes (DEGs) were identified using DESeq2, with thresholds of |log_2_(fold change)| greater than 1 and adjusted *p*-value less than 0.05.

Kyoto Encyclopedia of Genes and Genomes (KEGG) pathway enrichment analysis of the identified DEGs was conducted using the clusterProfiler (v4.6.2) R package. Terms with an adjusted *p*-value < 0.05 were considered significantly enriched.

### 2.12. Western Blot Analysis

Protein samples were prepared from both skin tissues and cultured HaCaT cells for Western blot analysis. Skin tissue samples were homogenized in RIPA lysis buffer supplemented with protease and phosphatase inhibitors (Servicebio, Wuhan, China; Cat. No. G2002) using a tissue homogenizer, followed by ultrasonic disruption. HaCaT cells, after respective treatments, were washed with cold PBS and lysed directly on ice using the same RIPA lysis buffer containing inhibitors for 30 min. The homogenates (from tissues) and lysates (from cells) were then centrifuged at 12,000× *g* for 15 min at 4 °C, and the supernatants were collected. Protein concentration was determined using a bicinchoninic acid (BCA) assay kit (Beyotime, Shanghai, China; Cat. No. P0012) according to the manufacturer’s instructions. Equal amounts of protein were separated by 10% sodium dodecyl sulfate-polyacrylamide gel electrophoresis (SDS-PAGE) and transferred onto polyvinylidene difluoride (PVDF) membranes (Millipore, USA). The membranes were blocked with 5% non-fat milk or 3% bovine serum albumin (BSA) in Tris-buffered saline containing 0.1% Tween 20 (TBST) for 1 h at room temperature and incubated overnight at 4 °C with primary antibodies against FGFR (ABclonal, Cat. No. A21219, 1:1000), p-FGFR (ABclonal, Cat. No. AP1317, 1:1000), PI3K (Affinity, Cat. No. AF6241, 1:1000), p-PI3K (Affinity, Cat. No. AF3241, 1:1000), AKT (ABclonal, Cat. No. A11016, 1:1000), p-AKT (ABclonal, Cat. No. AP1208, 1:1000), mTOR (ABclonal, Cat. No. A25581, 1:1000), p-mTOR (ABclonal, Cat. No. AP0115, 1:1000), p70S6K (ABclonal, Cat. No. A2190, 1:1000), and p-p70S6K (ABclonal, Cat. No. AP0502, 1:1000). After washing with TBST (3 × 10 min), membranes were incubated with a horseradish peroxidase (HRP)-conjugated goat anti-rabbit IgG secondary antibody (ABclonal, 1:5000) for 1 h at room temperature. Protein bands were visualized using an enhanced chemiluminescence (ECL) substrate (Millipore, Burlington, MA, USA) and imaged with a ChemiDoc XRS+ imaging system (Bio-Rad, Hercules, CA, USA). Band intensities were quantified using ImageJ (NIH) software.

### 2.13. RT-qPCR Assay

Total RNA was extracted from skin tissues and HaCaT cells using the M5 Universal RNA Rapid Extraction Kit (Mei5 Biotechnology, Beijing, China; Cat. No. MF787-01). The concentration and purity of the isolated RNA were evaluated using a BioSpectrometer basic spectrophotometer (Eppendorf, Hamburg, Germany). First-strand cDNA was synthesized from 1 µg of total RNA using the TransScript One-Step gDNA Removal and cDNA Synthesis SuperMix (TransGen Biotech, Beijing, China; Cat. No. AT311-02) in accordance with the manufacturer’s instructions.

qPCR was performed using a LightCycler 96 Real-Time PCR System (Roche, Basel, Switzerland). Each 20 µL reaction mixture consisted of 10 µL of 2× M5 HiPer SYBR Premix EsTaq, 2 µL of cDNA template, 0.8 µL (10 µM) of each forward and reverse primer, and 6.4 µL of nuclease-free water. The thermal cycling conditions included an initial denaturation at 94 °C for 30 s, followed by 40 cycles of denaturation at 94 °C for 5 s and annealing and extension at 60 °C for 30 s. The primer sequences used for quantitative analysis are listed in Table 1.

### 2.14. In Vitro Mechanistic Study with FGFR Inhibitor

To investigate the role of the FGFR/AKT signaling pathway in the protective effects of CP-L nanogel, a specific FGFR inhibitor, PD173074 (Beyotime Biotechnology, Shanghai, China; Cat. No. SC1045), was employed. HaCaT cells were randomly assigned to five experimental groups: (1) Control (no UVB, no treatment); (2) Model (UVB irradiation only); (3) CP-L nanogel (UVB irradiation + CP-L-TFs thermosensitive nanogel); (4) PD173074 (UVB irradiation + 10 μM PD173074); (5) CP-L nanogel + PD 173074 (UVB irradiation + CP-L nanogel + 10 μM PD173074). Cells were subjected to UVB irradiation (240 mJ/cm^2^) as described in Section 2.6. Immediately after irradiation, the medium was replaced with fresh complete medium containing the respective treatments (CP-L nanogel and/or PD173074). After 24 h of incubation, cell viability was assessed using the CCK-8 assay. For Western blot analysis, cells were lysed after the same treatment period, and the protein expression and phosphorylation levels of FGFR, p-FGFR, AKT, and p-AKT were analyzed according to the procedures described in Section 2.11.

### 2.15. Cellular Uptake Assay by Flow Cytometry

The hydrophobic fluorescent probe Coumarin 6 (C6, Shanghai Aladdin Biochemical Technology Co., Ltd., Shanghai, China; Cat. No. C100929) was used as a model drug to simulate the lipophilic components. Two formulations were prepared: Free C6 and C6-loaded Thermosensitive Nanogel (C6 nanogel). The C6 nanogel was fabricated using the identical method described for CP-L nanogel (Section 2.2), with C6 replacing CP-L during the preparation of transferosomes. The final C6 concentration in both formulations was calibrated to 1 µg/mL for cellular uptake studies. HaCaT cells were seeded in 6-well plates. After 24 h of attachment, cells were divided into three groups: (1) Control group; (2) Free C6 group; (3) C6 nanogel group. Cells were incubated with the respective formulations (containing 1 µg/mL C6) in serum-free medium at 37 °C for 4 h. Subsequently, the medium was aspirated, and cells were washed three times with cold PBS to thoroughly remove extracellular fluorescence. Cells were then harvested by trypsinization, centrifuged, and resuspended in 500 µL of PBS. The intracellular fluorescence intensity of at least 10,000 single-cell events per sample was immediately analyzed using a flow cytometer. The fluorescence was excited by a 488 nm laser and collected with a 525/40 nm bandpass filter (FL1 channel). Data were analyzed using FlowJo software (version 11.2), and the geometric MFI of the cell population was recorded for statistical comparison.

### 2.16. Molecular Docking and Dynamics Simulation

The three-dimensional crystal structures of the target proteins FGFR1 (PDB ID: 3RHX) and AKT1 (PDB ID: 4GV1) were retrieved from the Protein Data Bank (PDB, https://www.rcsb.org/). Proteins were prepared by removing water molecules and co-crystallized ligands, adding hydrogen atoms, and assigning appropriate protonation states at pH 7.4. The three-dimensional structures of the candidate CP-L compounds were obtained from the PubChem database and energetically minimized. Molecular docking was performed using AutoDock Vina (version 1.2.3). The binding sites were defined based on the co-crystallized ligands or key residues known from literature. A grid box of sufficient size encompassed the entire binding pocket. For each ligand-protein pair, multiple docking runs were conducted, and the conformation with the most favorable (lowest) binding affinity (ΔG, kcal/mol) was selected for subsequent analysis. Docking results were summarized in a heatmap, and key binding poses were visualized and analyzed for interactions (hydrogen bonds, hydrophobic contacts) using PyMOL (version 2.5.0).

To further evaluate the stability of the predicted ligand-protein complexes and to calculate the binding free energy more rigorously, molecular dynamics simulations were conducted using GROMACS (version 2025.1). The simulation systems were prepared by parameterizing the small-molecule ligands with the GAFF2 force field, while the protein structures were described using the AMBER14SB force field. Each complex was solvated in a cubic box of SPC/E water molecules, and the system was neutralized with counter-ions. The simulation protocol comprised two equilibration phases: first, 100 ps of NVT ensemble equilibration at 298 K, followed by 100 ps of NPT ensemble equilibration at 1 bar. Subsequently, a production MD run of 100 ns was carried out for each system, with trajectory snapshots saved every 10 ps for analysis. Finally, the binding free energy between each ligand and the corresponding protein was quantitatively estimated using the Molecular Mechanics Generalized Born Surface Area (MM/GBSA) method on frames extracted from the stable simulation trajectory.

### 2.17. Statistical Analysis

All data are expressed as the mean ± standard deviation (SD). The sample size (n) for each experiment is specified in the corresponding figure legends. Statistical analyses were performed using one-way analysis of variance (ANOVA) followed by least significant difference (LSD) post hoc tests for multiple group comparisons (SPSS software, version 27.0). Differences were considered statistically significant at *p* < 0.05.

## 3. Results

### 3.1. Chemical Compositions of CP-L

**Figure 1:**
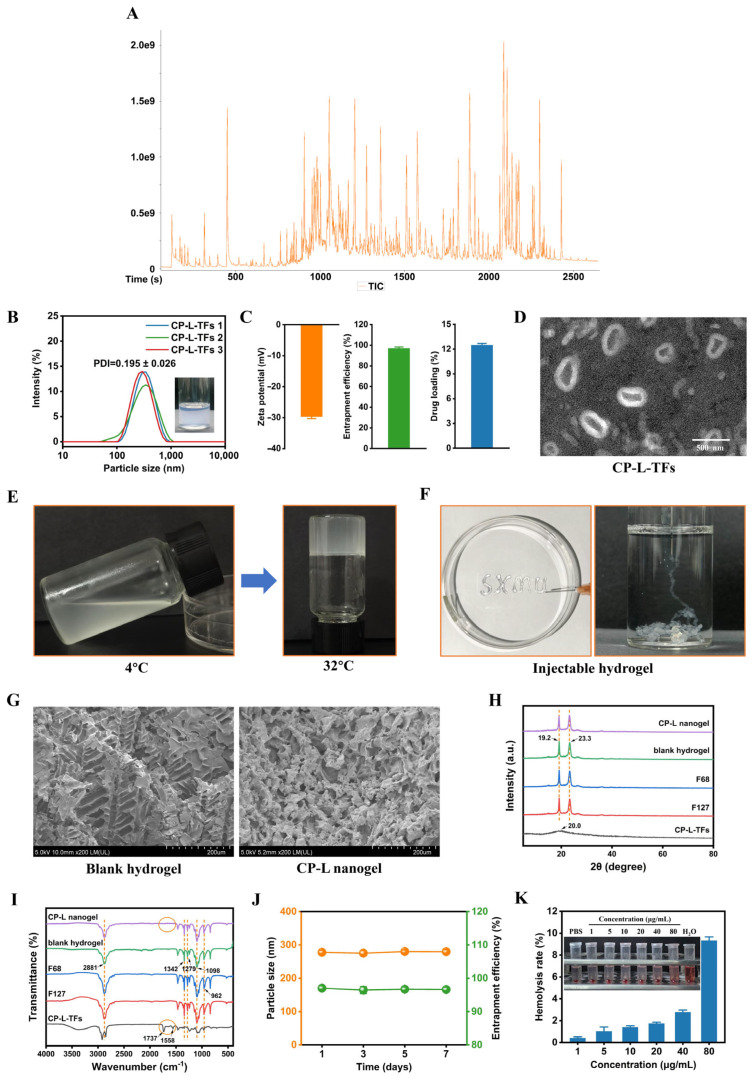
Characterization of CP-L extract and the developed thermosensitive nanogel system. (**A**) TIC of CP-L components. (**B**) Particle size distribution and appearance of CP-L-TFs. (**C**) Zeta potential, entrapment efficiency and drug loading of CP-L-TFs. (**D**) TEM image of CP-L-TFs (scale bar: 200 nm). (**E**) Sol to gel transition behavior of CP-L nanogel in response to temperature changes. (**F**) Injectability and deformability of CP-L nanogel. (**G**) Scanning electron microscopy images of blank hydrogel and CP-L nanogel. (**H**) XRD diffractograms of CP-L-TFs, Pluronic F127, Pluronic F68, blank hydrogel, and CP-L nanogel. (**I**) Fourier transform infrared spectra of CP-L-loaded transferosomes, Pluronic F127, Pluronic F68, blank hydrogel, and CP-L nanogel. (**J**) Storage stability of CP-L-TFs after 7 days of storage at 4 °C. (**K**) Hemolysis rates of CP-L nanogel. Data in panels (**B**,**C**,**J**,**K**) are presented as mean ± SD (n = 3 independent preparations).

The chemical constituents of CP-L were characterized by gas chromatography mass spectrometry analysis. A representative total ion chromatogram (TIC) of the quality control sample is presented in Figure 1A. Compound identification was achieved by matching mass spectral data and retention indices with the National Institute of Standards and Technology database, and relative abundances were quantified based on chromatographic peak areas. As summarized in Table 2, the major constituents included ethyl linoleate [35,36], paeonol [37,38], muscone [39,40], decanoic acid [41,42], and palmitic acid. These compounds have been reported to possess various biological activities and were therefore considered the primary bioactive constituents of CP-L.

### 3.2. Characterization of the CP-L-TFs Thermosensitive Nanogel System

The lipophilic components extracted from *Codonopsis pilosula* were successfully encapsulated into TFs through hydrophobic interactions. Unlike conventional liposomes, CP-L-TFs were prepared using phospholipids and NaDC, a bile salt functioning as a membrane-softening agent. As shown in Figure 1B, the resulting CP-L-TFs exhibited a bluish opalescent appearance under visible light. Dynamic light scattering analysis demonstrated that the CP-L-TFs had a mean particle size of 276.90 ± 11.27 nm, a *PDI* of 0.195 ± 0.026, and a zeta potential of −29.70 ± 0.61 mV (Figure 1B,C). The entrapment efficiency and drug loading were calculated to be 97.11% ± 1.22% and 12.51 ± 0.17% (Figure 1C), indicating efficient encapsulation of CP-L within the vesicles. TEM images revealed that the CP-L-TFs exhibited an elliptical morphology with an average diameter of approximately 300 nm (Figure 1D), consistent with the dynamic light scattering results and indicative of vesicle deformability. As shown in Figure 1J, after 7 days of storage, the changes in particle size and EE for CP-L-TFs were all less than 5%, indicating that CP-L-TFs was stable for 7 days at 4 °C.

A thermosensitive nanogel formulation was subsequently developed by incorporating CP-L-TFs into a mixed Pluronic F127 and F68 matrix. The suitability of the formulation for topical application was evaluated based on its thermosensitive behavior, injectability, and deformability. A reversible sol to gel transition was observed at 32 °C (Figure 1E), corresponding to the temperature of the human skin surface and enabling in situ gelation after application. Injectability was demonstrated by the smooth extrusion of the gel precursor through a syringe into distilled water maintained at 32 °C, where the formulation retained its structural integrity (Figure 1F). Deformability was further confirmed by the successful formation of the letters “SXMU” during extrusion (Figure 1F).

Scanning electron microscopy analysis revealed that the blank hydrogel exhibited a smooth cross-section with a continuous and uniformly distributed porous structure (Figure 1G). In contrast, the CP-L nanogel displayed a rougher cross-section with reduced pore size, demonstrating that incorporation of the TFs altered the microstructure of the thermosensitive hydrogel. XRD and FT-IR were employed to investigate potential interactions between CP-L-TFs and the hydrogel matrix. The XRD diffractograms of CP-L-TFs, Pluronic F127, Pluronic F68, blank hydrogel, and CP-L nanogel are presented in Figure 1H. CPL-TFs exhibited an amorphous structure, characterized by a broad peak near 20°. In contrast, Pluronic F127, Pluronic F68, and blank hydrogel displayed two characteristic sharp diffraction peaks at 19.2° and 23.3°. After the incorporation of CP-L-TFs into blank hydrogel, the intensity of the characteristic peak at 19.2° slightly decreased, while no other changes were observed, indicating successful encapsulation of CP-L-TFs within the hydrogel. The FT-IR spectra of Pluronic F127, Pluronic F68, and blank hydrogel exhibited identical characteristic absorption peaks at 2881, 1342, 1279, 1098, and 962 cm^−1^, confirming the composition of the blank hydrogel (Figure 1I). In the spectrum of CP-L nanogel, characteristic peaks originating from CP-L-TFs, such as the carbonyl stretching vibration at 1737 cm^−1^ and the N–H bending vibration at 1558 cm^−1^, were markedly attenuated or absent, indicating successful embedding of the vesicles within the hydrogel matrix. As shown in Figure 1K, red blood cells were incubated with varying concentrations of CP-L nanogel (1, 5, 10, 20, 40, and 80 μg/mL) for 3 h. The hemolysis rates remained below 3% across the concentration range of 1–40 μg/mL, demonstrating good blood compatibility at these tested doses.

### 3.3. Changes in Skin Appearance of Photoaged Mice

**Figure 2:**
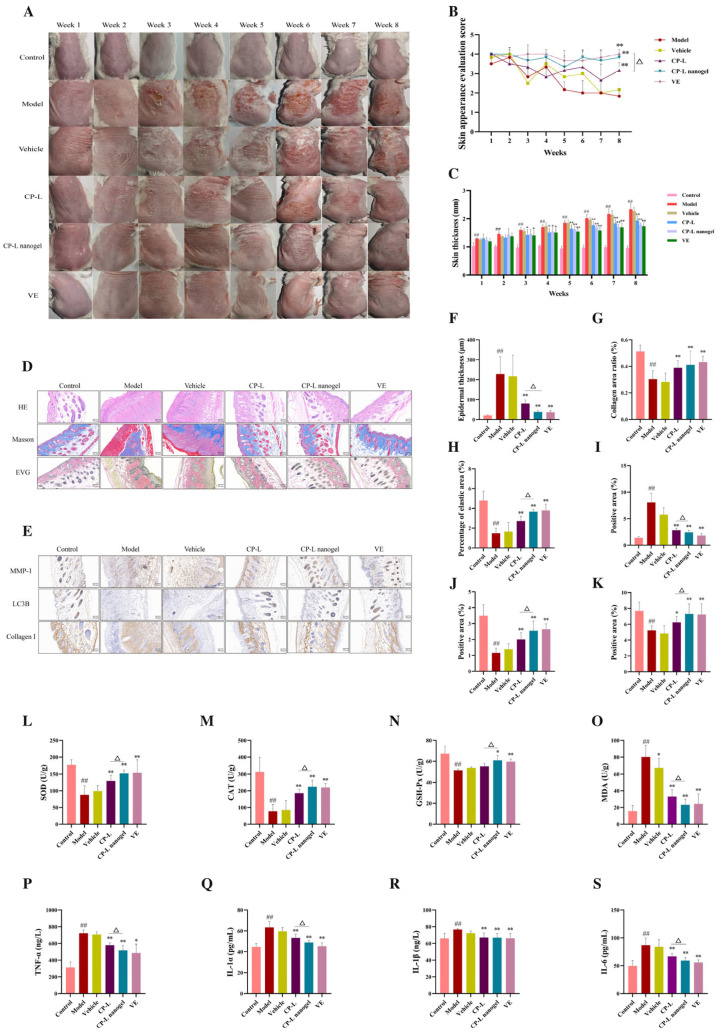
CP-L nanogel attenuated UV-induced skin photoaging in mice. (**A**) Representative photographs of mouse dorsal skin appearance after 8 weeks of treatment. (**B**) Quantitative skin condition scores. (**C**) Measurement of skin thickness. (**D**) Representative images of skin tissue sections stained with H&E, Masson’s trichrome, and EVG. (**E**) Representative IHC staining images for MMP-1, LC3B, and Collagen I. (**F**) Epidermal thickness. (**G**) Collagen area ratio. (**H**) Percentage of elastic fiber area. (**I**–**K**) Quantitative analysis of IHC positive staining area for (**I**) MMP-1, (**J**) LC3B, and (**K**) Collagen I. (**L**–**O**) Levels of oxidative stress markers in skin tissue: (**L**) SOD, (**M**) CAT, (**N**) GSH-Px, and (**O**) MDA. (**P**–**S**) Concentrations of pro-inflammatory cytokines: (**P**) TNF-α, (**Q**) IL-1α, (**R**) IL-1β, and (**S**) IL-6. Data are presented as mean ± SD (n = 6 for in vivo data; n = 3 for IHC quantification from independent experiments). Statistical significance was determined by one-way ANOVA followed by LSD post hoc tests. ## *p* < 0.01 vs. control group; * *p* < 0.05, ** *p* < 0.01 vs. the model group; △ *p* < 0.05 vs. CP-L group.

Chronic UV irradiation induced significant photoaging damage, as evidenced by pronounced dorsal skin dryness, deep wrinkle formation, erythema, and loss of elasticity in the Model group compared to the smooth and intact skin of the Control group (Figure 2A). Topical treatment with unformulated CP-L moderately alleviated these symptoms. Notably, the CP-L nanogel demonstrated the most substantial improvement, restoring skin appearance closest to the normal state among all intervention groups. The positive group (VE) also showed a clear improvement in skin condition compared to the Model group (Figure 2A).

Consistent with the visual observations, quantitative skin condition scoring (based on the criteria in Table 3) revealed that both CP-L and CP-L nanogel treatments significantly reduced the photoaging scores compared to the Model group (Figure 2B). The therapeutic effect of the CP-L nanogel was significantly more pronounced than that of unformulated CP-L (Figure 2B), underscoring the advantage of the nano-formulation. Treatment with VE similarly resulted in a significant reduction in the photoaging score relative to the Model group.

Histometric analysis further demonstrated that chronic UV exposure led to a significant increase in epidermal thickness, a hallmark of photoaging pathology. Both CP-L and CP-L nanogel treatments effectively attenuated this UV-induced epidermal hyperplasia (Figure 2C). Similar to the macroscopic and scoring results, the CP-L nanogel exhibited a superior inhibitory effect on epidermal thickening compared to the unformulated extract (Figure 2C). Epidermal thickness was also significantly reduced in the VE-treated group compared to the Model group.

### 3.4. Histopathological Assessment of Photoaged Skin

Histopathological evaluation via H&E staining confirmed significant UV-induced epidermal hyperplasia in the Model group compared to the well-organized, thin epidermis of the Control group (Figure 2F). Treatments with CP-L, CP-L nanogel, and VE all markedly attenuated this hyperplasia (*p* < 0.01 vs. Model group), with the nanogel group showing a more pronounced restoration of normal skin architecture (*p* < 0.05 vs. CP-L group; Figure 2F).

Masson’s trichrome staining, which selectively stains collagen fibers blue, further demonstrated severe collagen degradation in UV exposed skin (Figure 2G). Compared with the dense and well bundled collagen network observed in the control group, the model group exhibited fragmented and sparse collagen deposition (*p* < 0.01 vs. control group). In contrast, CP-L, CP-L nanogel, and VE treatments all significantly restored collagen density and structural integrity (*p* < 0.01 vs. model group).

EVG staining revealed a substantial reduction in black stained elastic fibers in the model group. CP-L, CP-L nanogel, and VE treatments promoted the recovery of elastic fiber content to varying extents (Figure 2H, *p* < 0.01 vs. model group), with the CP-L nanogel demonstrating a superior effect compared to CP-L alone (*p* < 0.05 vs. CP-L group).

IHC provided further mechanistic insights (Figure 2I–K). Expression of the matrix-degrading enzyme MMP-1 was significantly upregulated in the Model group (*p* < 0.01 vs. Control; Figure 2I). Conversely, staining for the autophagy marker LC3B was diffuse and weak, while Collagen I staining appeared sparse and fragmented in the Model group (Figure 2J,K). Treatment with CP-L, CP-L nanogel, and VE markedly downregulated MMP-1 expression (*p* < 0.01 vs. Model group; Figure 2I). Notably, LC3B staining exhibited a distinct punctate pattern following treatment, suggesting the restoration of autophagic flux (Figure 2J). Concurrently, Collagen I expression was substantially improved, as evidenced by increased staining intensity and fiber density, with the CP-L nanogel again showing superior efficacy (*p* < 0.05 vs. CP-L group; Figure 2K).

### 3.5. Restoration of Antioxidant Defenses and Suppression of Inflammation by CP-L Nanogel In Vivo

As shown in Figure 2L–O, compared to the Model group, treatment with unformulated CP-L, CP-L nanogel, and VE significantly increased the activities of key antioxidant enzymes, including SOD, CAT, and GSH-Px (*p* < 0.05 or *p* < 0.01), while markedly reducing the level of the lipid peroxidation product MDA (*p* < 0.01). Importantly, the CP-L nanogel exhibited a significantly stronger restorative effect on these oxidative stress markers than unformulated CP-L (*p* < 0.05).

Furthermore, UV exposure also triggered a potent pro-inflammatory response, evidenced by significantly elevated levels of cytokines including TNF-α, IL-1α, IL-1β, and IL-6 in the Model group (Figure 2P–S). Treatment with CP-L, CP-L nanogel, and VE effectively downregulated the expression of these inflammatory mediators at the protein level (*p* < 0.01 vs. Model group). Consistent with the antioxidant profile, the anti-inflammatory efficacy of the CP-L nanogel was significantly superior to that of unformulated CP-L (*p* < 0.05).

### 3.6. Transcriptomic Analysis Identifies the PI3K-AKT Pathway as a Key Target in Photoaged Skin

**Figure 3:**
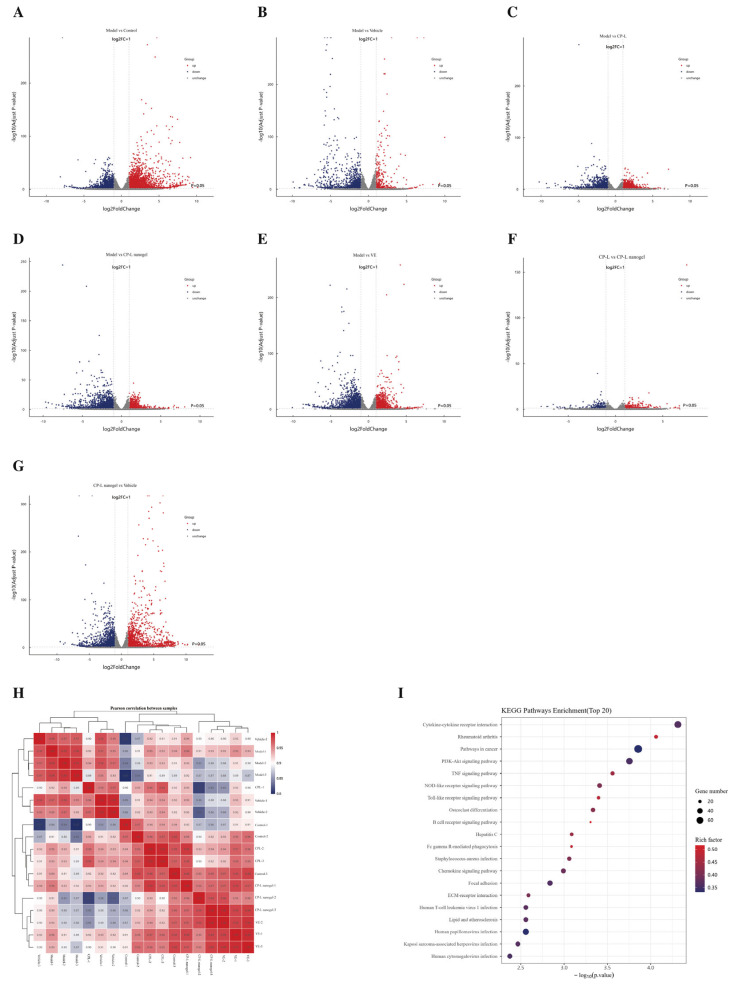
Transcriptomic profiling of DEGs. (**A**–**F**) Volcano plots of paired comparisons: (**A**) model vs. control, (**B**) model vs. vehicle, (**C**) model vs. CP-L, (**D**) model vs. CP-L nanogel, (**E**) model vs. VE, and (**F**) CP-L vs. CP-L nanogel. (**G**) CP-L nanogel vs. vehicle. (**H**) Heatmap of differentially expressed gene expression patterns across groups. (**I**) KEGG pathway enrichment analysis of genes differentially expressed between the CP-L and model groups and between the CP-L nanogel and model groups. DEGs were identified with thresholds of |log_2_(fold change)| > 1 and adjusted *p*-value < 0.05 (DESeq2).

High throughput transcriptomic analysis was conducted to investigate gene expression changes in skin tissues from the control, model, vehicle, CP-L, CP-L nanogel, and VE groups (Figure 3). Compared with the control group, the model group exhibited 4639 DEGs, including 3051 upregulated and 1588 downregulated genes. Relative to the model group, the number of DEGs in each treatment group was as follows: vehicle group, 2335 genes (650 upregulated and 1685 downregulated); CP-L group, 3314 genes (1241 upregulated and 2073 downregulated); CP-L nanogel group, 3485 genes (1221 upregulated and 2264 downregulated); and VE group, 4164 genes (1445 upregulated and 2719 downregulated). In addition, the CP-L nanogel group exhibited 1275 DEGs compared with the CP-L group (625 upregulated and 650 downregulated) and 4089 DEGs compared with the vehicle group (2068 upregulated and 2021 downregulated).

KEGG enrichment analysis of the DEGs reversed by CP-L and CP-L nanogel treatments identified several significantly modulated pathways. Most prominently, the PI3K-AKT signaling pathway was significantly enriched (Figure 3I), suggesting its central role in the protective mechanism against photoaging. Given the critical upstream position of FGFRs in activating the PI3K-AKT pathway and their documented involvement in UV-induced skin responses, we focused subsequent mechanistic validation on this specific signaling axis.

### 3.7. Western Blot Analysis Revealed Suppression of the FGFR/PI3K/AKT/mTOR/p70S6K Axis by CP-L Nanogel In Vivo

**Figure 4:**
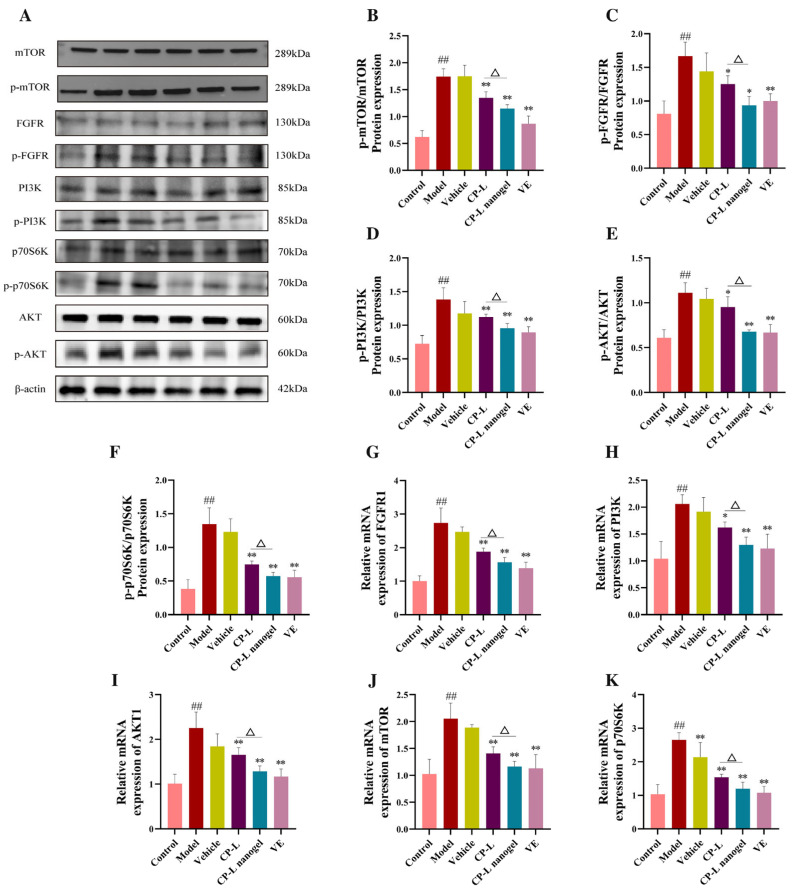
CP-L nanogel inhibited the activation of the FGFR/PI3K/AKT/mTOR pathway in photoaged mouse skin. (**A**) Representative Western blot images showing the expression and phosphorylation levels of key proteins in the FGFR/PI3K/AKT/mTOR/p70S6K axis. (**B**–**F**) Quantitative analysis of protein phosphorylation levels, expressed as the ratio of phosphorylated to total protein for (**B**) mTOR, (**C**) FGFR, (**D**) PI3K, (**E**) p70S6K, and (**F**) AKT. (**G**–**K**) Relative mRNA expression levels of pathway-related genes determined by RT-qPCR: (**G**) FGFR1, (**H**) PI3K, (**I**) AKT1, (**J**) mTOR, and (**K**) p70S6K. Data are presented as mean ± SD (n = 3 for Western blot quantification from independent experiments; n = 6 mice per group for RT-qPCR). Statistical significance was determined by one-way ANOVA followed by LSD post hoc tests. ## *p* < 0.01 vs. Control group; * *p* < 0.05, ** *p* < 0.01 vs. Model group; △ *p* < 0.05 vs. CP-L group.

Western blot analysis demonstrated that chronic UV irradiation significantly increased the phosphorylation levels of key proteins in the FGFR–PI3K–AKT–mTOR–p70S6K signaling axis, including FGFR, PI3K, AKT, mTOR, and p70S6K, compared with the control group, whereas the total protein levels of these molecules remained unchanged (Figure 4). Treatments with CP-L, CP-L nanogel, and the positive control (VE) all effectively reversed this UV-induced hyperphosphorylation (*p* < 0.05 or *p* < 0.01 vs. model group). Notably, the inhibitory effect was significantly more pronounced in the CP-L nanogel group than in the CP-L group (*p* < 0.05).

### 3.8. RT-qPCR Analysis of Pathway-Related Gene Expression

RT-qPCR analysis confirmed that chronic UV irradiation significantly upregulated the mRNA expression of key components of the FGFR–PI3K–AKT–mTOR–p70S6K signaling pathway, including FGFR, PI3K, AKT, mTOR, and p70S6K, in photoaged skin compared with normal controls (Figure 4). Treatment with CP-L, CP-L nanogel, and the positive control (VE) all effectively reversed these transcriptional alterations, significantly downregulating the expression of these genes (*p* < 0.01 or *p* < 0.05 vs. model group), with a more pronounced inhibitory effect observed in the CP-L nanogel group (*p* < 0.05 vs. CP-L group).

### 3.9. CP-L Nanogel Protected HaCaT Keratinocytes from UVB-Induced Cytotoxicity

**Figure 5:**
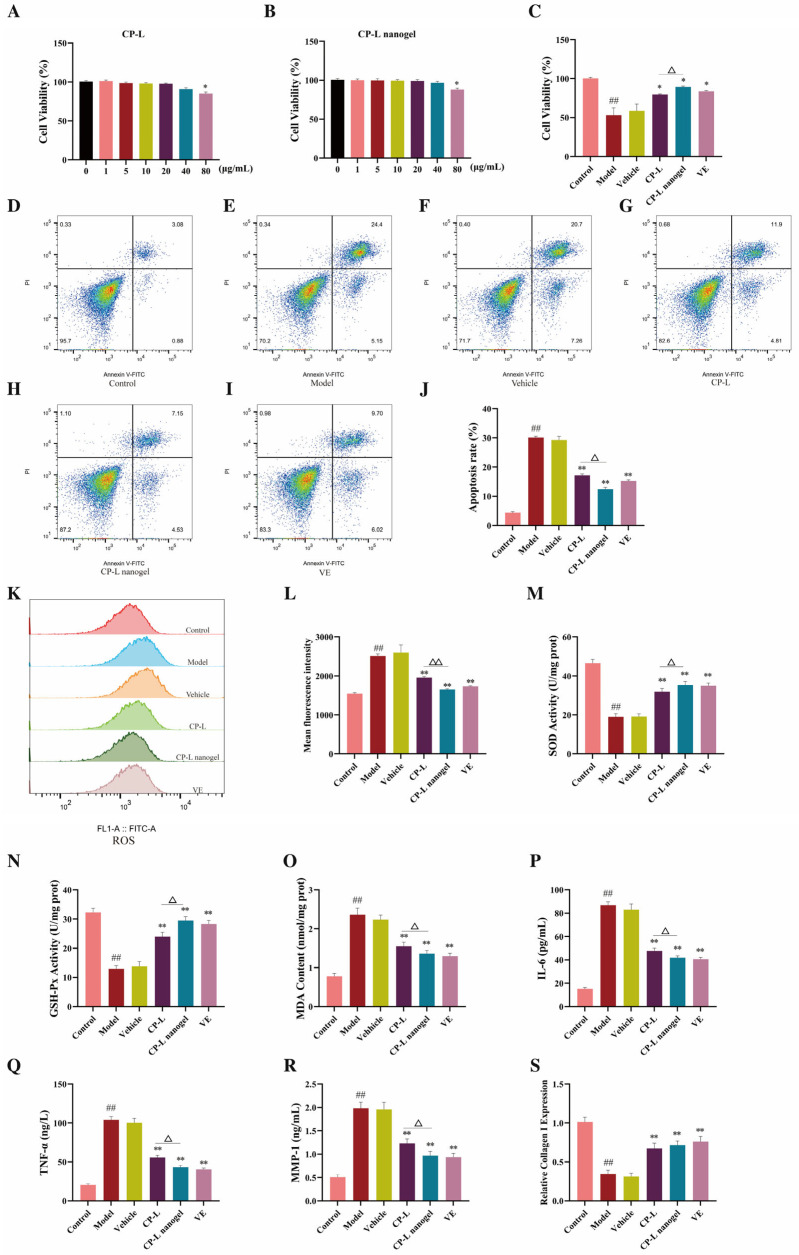
Protective effects of CP-L nanogel against UVB-induced damage in HaCaT keratinocytes. (**A**) Cell viability of HaCaT cells treated with various concentrations of unformulated CP-L (0–80 μg/mL) for 24 h without UVB irradiation, assessed by CCK-8 assay. (**B**) Cell viability of HaCaT cells treated with various concentrations of CP-L nanogel (0–80 μg/mL) for 24 h without UVB irradiation, assessed by CCK-8 assay. (**C**) Cell viability of HaCaT cells after UVB irradiation and post-treatment with the indicated formulations, assessed by CCK-8 assay. (**D**–**I**) Flow cytometry plots of Annexin V-FITC/PI staining showing apoptotic cell distribution: (**D**) Control, (**E**) Model, (**F**) Vehicle Gel, (**G**) CP-L, (**H**) CP-L nanogel, and (**I**) Positive. (**J**) Quantitative analysis of total apoptotic cell rate (early + late apoptotic) derived from (**D**–**I**). (**K**) Representative flow cytometry histograms of intracellular ROS levels detected by DCFH-DA fluorescence. (**L**) MFI of ROS. (**M**–**O**) Levels of oxidative stress markers in cell lysates: (**M**) SOD, (**N**) GSH-Px, and (**O**) MDA. (**P**–**S**) Concentrations of pro-inflammatory mediators in cell culture supernatants: (**P**) IL-6, (**Q**) TNF-α, (**R**) MMP-1, and (**S**) Collagen I. Data are presented as mean ± SD (n = 6). Statistical significance was determined by one-way ANOVA followed by LSD post hoc tests. ## *p* < 0.01 vs. Control group; * *p* < 0.05, ** *p* < 0.01 vs. Model group; △ *p* < 0.05, △△ *p* < 0.05 vs. CP-L group.

The cytotoxic effect of UVB irradiation and the protective efficacy of various treatments were evaluated by CCK-8 assay in HaCaT cells. Preceding the main study, a dose–response experiment confirmed that both unformulated CP-L and CP-L nanogel (0–40 μg/mL) showed no significant cytotoxicity. Based on this, a concentration of 20 μg/mL (CP-L equivalent) was selected for subsequent protective studies. As shown in Figure 5C, a single UVB irradiation significantly reduced the viability of HaCaT cells to 53.0 ± 9.6% relative to the non-irradiated Control group (set as 100%, *p* < 0.01), successfully establishing the photoaging damage model. Treatment with the blank Vehicle Gel following UVB exposure did not improve cell viability (58.6 ± 8.7%, *p* > 0.05 vs. Model group), indicating the hydrogel carrier itself lacked protective activity.

In contrast, post-UVB treatment with unformulated CP-L significantly attenuated cell damage, increasing viability to 79.5 ± 0.8% (*p* < 0.05 vs. Model group). The CP-L nanogel demonstrated a superior protective effect, restoring cell viability to 89.4 ± 1.0%, which was significantly higher than that of the CP-L group (*p* < 0.05). The positive group treated with VE showed a comparable protective effect, with viability at 83.8 ± 1.0% (*p* < 0.05 vs. Model group).

### 3.10. CP-L Nanogel Ameliorated UVB-Induced Apoptosis in HaCaT Keratinocytes

The protective effect against UVB-induced apoptosis was quantitatively evaluated using Annexin V-FITC/PI staining. As shown in Figure 5J, chronic UVB exposure markedly induced apoptosis in HaCaT cells. Compared to the control group (total apoptotic cells: 4.37 ± 0.37%), the model group exhibited a significant increase in total apoptosis (30.1 ± 0.48%, *p* < 0.01). Treatment with the blank Vehicle Gel did not attenuate this effect (29.27 ± 1.27%, *p* > 0.05), confirming that the hydrogel base itself was inactive. Post-UVB treatment with unformulated CP-L significantly reduced the apoptotic rate to 17.14 ± 0.50% (*p* < 0.05). The CP-L nanogel demonstrated the most potent anti-apoptotic activity, lowering the total apoptosis to 12.44 ± 0.66%, which was significantly lower than the CP-L group (*p* < 0.05) and closely approximated the level of the Control group. The Positive group (VE) also showed a strong protective effect (15.24 ± 0.42%, *p* < 0.01).

### 3.11. CP-L Nanogel Attenuated UVB-Induced Intracellular ROS Generation

Intracellular ROS levels were quantified by flow cytometry using the DCFH-DA probe to evaluate the antioxidative capacity of the formulations. As shown in Figure 5L, UVB irradiation triggered a sharp increase in ROS, with a significantly higher MFI compared to the Control group (*p* < 0.01).

Post-treatment with unformulated CP-L partially scavenged ROS, resulting in a significantly lower MFI than the Model group (*p* < 0.01). The positive control (VE) also significantly reduced ROS levels compared to the Model group (*p* < 0.01). Strikingly, the CP-L nanogel demonstrated superior efficacy, reducing the MFI to a level nearly equivalent to that of the Control group. This represented a highly significant reduction relative to the Model group (*p* < 0.01) and a statistically significant improvement over treatment with unformulated CP-L (*p* < 0.05).

### 3.12. CP-L Nanogel Mitigated Oxidative Stress and Inflammation in UVB-Irradiated HaCaT Cells

The effects of CP-L and its nano-formulation on UVB-induced oxidative stress and inflammation were evaluated in HaCaT cells (Figure 5M–S). UVB irradiation (model group) significantly induced oxidative damage, as evidenced by a marked decrease in the activities of antioxidant enzymes (SOD and GSH-Px) and a concurrent increase in the lipid peroxidation product (MDA) compared to the control group (*p* < 0.01). This was accompanied by a strong pro-inflammatory response, with significant upregulation of secreted IL-6 and TNF-α, as well as the matrix-degrading enzyme MMP-1, and a concomitant downregulation of collagen I synthesis (*p* < 0.01). Treatment with unformulated CP-L partially reversed these detrimental changes, showing significant improvements in all measured markers compared to the model group (*p* < 0.01). The positive control (VE) similarly demonstrated significant protective effects across these parameters (*p* < 0.01 vs. Model). However, the CP-L nanogel demonstrated a significantly superior protective effect. It effectively restored antioxidant enzyme activities, reduced MDA and pro-inflammatory cytokine levels, and notably downregulated MMP-1 while upregulating Collagen I synthesis. The efficacy of CP-L nanogel was significantly greater than that of the unformulated CP-L (*p* < 0.05). The Vehicle Gel group showed no protective effect.

### 3.13. CP-L Nanogel Downregulated the Expression of FGFR/PI3K/AKT/mTOR Pathway Genes in HaCaT Cells

**Figure 6:**
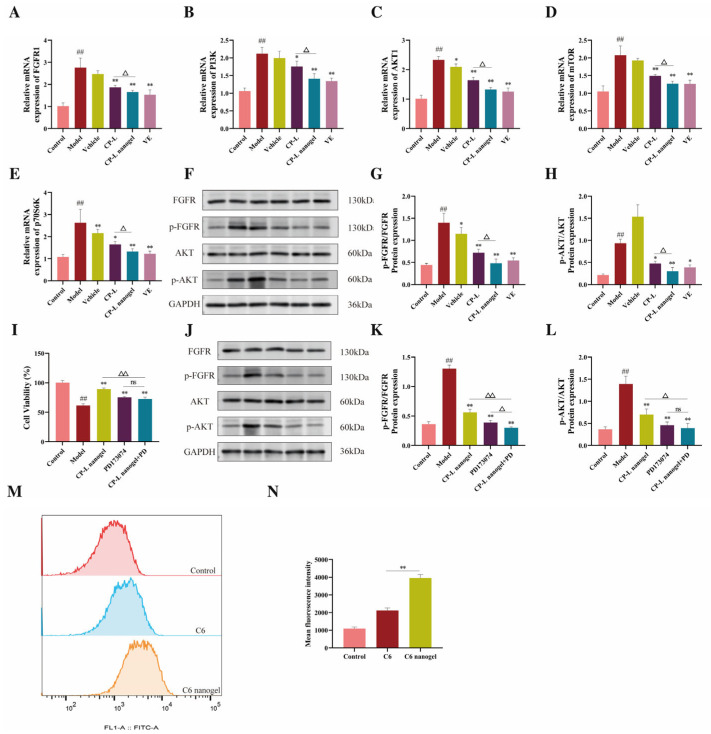
CP-L nanogel inhibited the FGFR/PI3K-AKT/mTOR pathway in HaCaT cells and its cytoprotection is FGFR-dependent. (**A**–**E**) Relative mRNA expression levels of key genes in the FGFR/PI3K-AKT/mTOR pathway determined by RT-qPCR: (**A**) FGFR1, (**B**) PI3K, (**C**) AKT1, (**D**) mTOR, and (**E**) p70S6K. (**F**) Representative Western blot images showing the protein expression and phosphorylation levels of FGFR and AKT. (**G**,**H**) Quantitative analysis of the phosphorylation levels, expressed as the ratio of phosphorylated to total protein for (**G**) FGFR and (**H**) AKT. (**I**) Cell viability assessed by CCK-8 assay after treatment with CP-L nanogel and/or the specific FGFR inhibitor PD173074 following UVB irradiation. (**J**) Representative Western blot images showing the protein expression and phosphorylation levels of FGFR and AKT in the presence of PD173074. (**K**,**L**) Quantitative analysis of the phosphorylation levels for (**K**) FGFR and (**L**) AKT under inhibitor treatment conditions. (**M**) Representative flow cytometry histograms of cellular uptake efficiency, comparing Free C6 and C6-loaded nanogel. (**N**) Quantitative analysis of the MFI. Data are presented as mean ± SD (n = 6 for RT-qPCR and cell viability; n = 3 for Western blot quantification and uptake assay, representing independent experiments). Statistical significance was determined by one-way ANOVA followed by LSD post hoc tests. ## *p* < 0.01 vs. Control group; * *p* < 0.05, ** *p* < 0.01 vs. Model group; △ *p* < 0.05, △△ *p* < 0.01 vs. CP-L nanogel group.

As presented in Figure 6A–E, UVB irradiation significantly upregulated the mRNA expression levels of FGFR1, PI3K, AKT1, mTOR and p70s6k in model group as compared to the control group (*p* < 0.01), indicating the activation of this signaling axis during photoaging damage. Treatment with unformulated CP-L significantly downregulated the UVB-induced overexpression of all five genes (*p* < 0.01 or *p* < 0.05 vs. Model). The positive control (VE) similarly reversed the upregulated gene expression (*p* < 0.01 vs. Model). The CP-L nanogel exhibited a more potent inhibitory effect, reducing the mRNA levels to a greater extent than the free CP-L treatment. The difference between the CP-L nanogel and CP-L groups was statistically significant for all genes (*p* < 0.05).

### 3.14. CP-L Nanogel Suppressed UVB-Induced Phosphorylation of FGFR and AKT in HaCaT Cells

Western blot analysis was performed to evaluate the effects of treatments on the activation status of the FGFR/AKT signaling axis in UVB-irradiated HaCaT cells (Figure 6G,H). The results focused on the phosphorylation levels of FGFR and AKT as indicators of pathway activity. Compared to the control group, UVB irradiation (model group) induced a significant increase in the phosphorylation levels of both FGFR and AKT (*p* < 0.01), while their total protein expression remained stable. This confirms the specific activation of this signaling pathway under photoaging stress. Post-UVB treatment with unformulated CP-L significantly attenuated the phosphorylation of both p-FGFR and p-AKT (*p* < 0.05 or *p* < 0.01 vs. Model group). VE similarly inhibited the UVB-induced phosphorylation of FGFR and AKT (*p* < 0.01 vs. Model). The CP-L nanogel exhibited a more pronounced inhibitory effect, reducing the phosphorylation levels significantly further than the free CP-L (*p* < 0.05).

### 3.15. FGFR/AKT Pathway Dependency of CP-L Nanogel Cytoprotection Validated by PD173074 Inhibition

To elucidate whether the FGFR/AKT pathway mediates the cytoprotection by CP-L nanogel, we employed the specific FGFR inhibitor PD173074. Both cell viability and Western blot analyses confirmed the functional reliance of CP-L nanogel on this axis. The protective effect of CP-L nanogel was entirely abolished upon co-treatment with PD173074. While CP-L nanogel alone significantly restored cell viability after UVB irradiation (*p* < 0.01), viability in the combination (CP-L nanogel + PD) group dropped to a level comparable to that of the inhibitor-alone group and was significantly lower than with CP-L nanogel alone (*p* < 0.01). PD173074 itself also conferred significant protection (*p* < 0.01 vs. CP-L nanogel, Figure 6I). Consistent with the functional readout, Western blot analysis revealed that PD173074 potently suppressed UVB-induced phosphorylation of FGFR and AKT (Figure 6K,L). In the combination group, the phosphorylation levels of p-FGFR and p-AKT mirrored those in the inhibitor-only group and remained significantly higher than after treatment with CP-L nanogel alone (*p* < 0.01). These data collectively indicated that pharmacological blockade of FGFR overrides the inhibitory effect of CP-L nanogel on this signaling cascade, confirming the functional reliance of its cytoprotection on the FGFR/AKT axis.

### 3.16. Enhanced Cellular Uptake of the CP-L Nanogel System Quantified by Flow Cytometry

Flow cytometric analysis of Coumarin 6 fluorescence provided direct evidence for the enhanced cellular delivery capability of the nanogel system. As shown in Figure 6, cells treated with C6 nanogel exhibited a dramatically higher fluorescence signal compared to those treated with Free C6. The geometric MFI of the C6 nanogel group was approximately 1.8-fold higher than that of the Free C6 group (*p* < 0.01, Figure 6N). The fluorescence histogram showed a marked rightward shift for the entire cell population in the C6 nanogel group, indicating a uniform and highly efficient uptake across the culture (Figure 6M).

### 3.17. Molecular Docking of CP-L Components with Key Proteins in the FGFR/PI3K-AKT/mTOR Pathway

**Figure 7:**
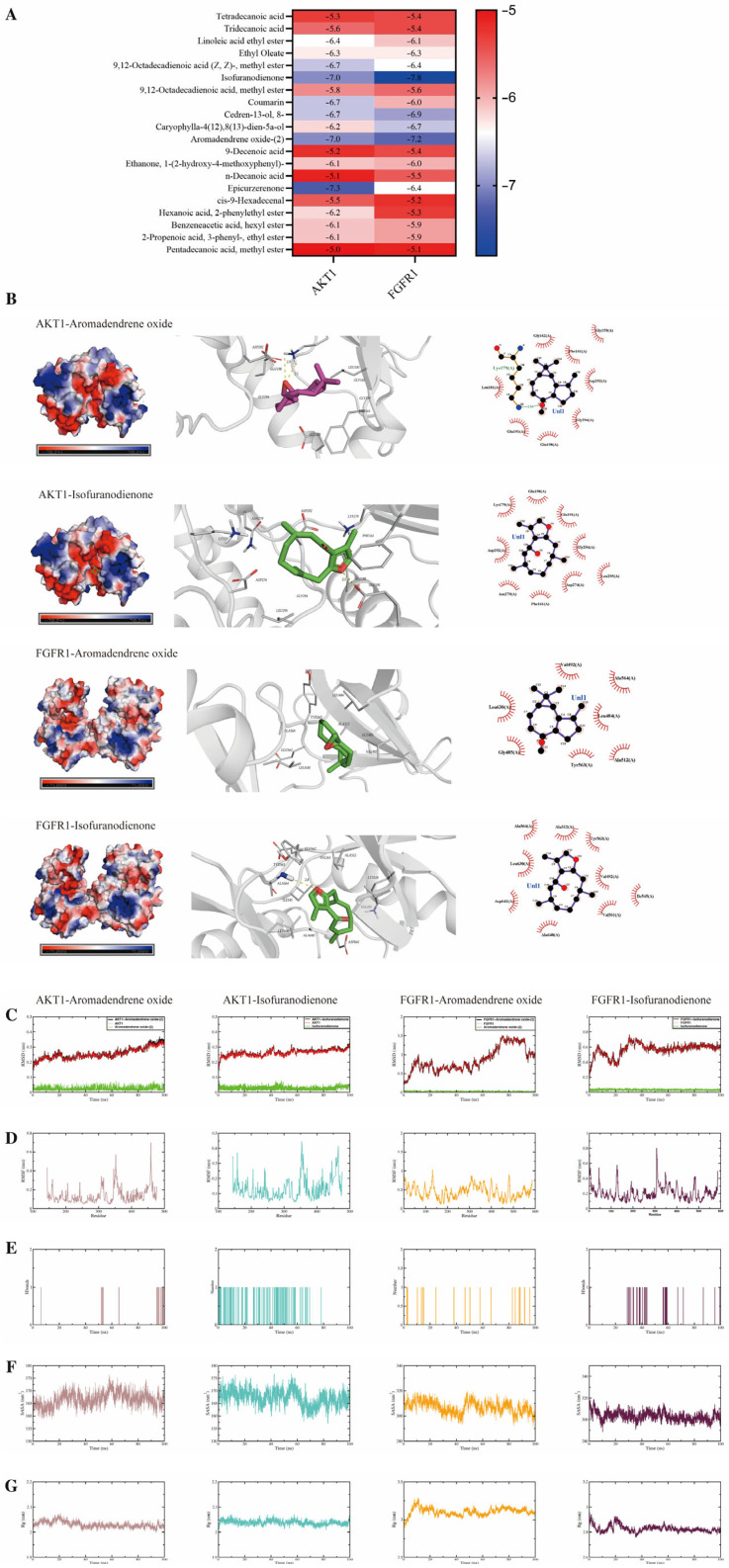
Computational analysis of the interaction between key CP-L constituents and target proteins. (**A**) Heatmap of molecular docking binding energies (kcal/mol) between 20 major constituents iof CP-L and the target proteins FGFR1 and AKT1. Lower binding energy indicates higher predicted affinity. (**B**) Representative three-dimensional binding poses from molecular docking, visualizing the predicted interaction modes of the top-ranked compounds (isofuranodienone and aromadendrene oxide-(2)) within the active sites of FGFR1 and AKT1. (**C**–**G**) Stability analysis of the top ligand-protein complexes from 100-ns MD simulations: (**C**) RMSD of the protein backbone and ligand atoms over time, reflecting overall structural stability. (**D**) RMSF per residue, indicating local flexibility, especially in binding site regions. (**E**) Number of intermolecular hydrogen bonds between the ligand and protein throughout the simulation. (**F**) SASA of the complex, monitoring changes in surface exposure. (**G**) Rg of the protein, assessing overall compactness and folding stability.

To verify the interactions between 20 major bioactive components of CP-L and two key targets (FGFR1 and AKT1) in the FGFR/PI3K-AKT/mTOR pathway, molecular docking experiments were performed. A lower binding energy indicates a more stable interaction between the ligand and the target protein. Typically, docking energies below −4.25 kcal/mol and −5.0 kcal/mol are considered to indicate binding activity and good binding activity, respectively. As shown in Figure 1, the docking energies of all 20 components with both proteins were below −5.0 kcal/mol, indicating that these major components of CP-L have the ability to regulate key proteins in this pathway. Notably, isofuranodienone and aromadendrene oxide-(2) exhibited particularly strong binding abilities, with docking energies below −7 kcal/mol for both targets. The docking conformations and key interacting residues of these two compounds with FGFR1 and AKT1 were visualized using PyMOL software (version 3.1.0; Figure 7B).

### 3.18. Molecular Dynamics (MD) Simulation

To further assess the stability of the top-ranked docking complexes under physiological conditions, MD simulations were conducted for four systems: arodendendrene oxide-(2)-AKT1, isofuranodienone-AKT1, aromadendrene oxide-(2)-FGFR1, and isofuranodienone-FGFR1. Each system was simulated for 100 ns, and the trajectories were analyzed to evaluate complex stability, flexibility, and interaction persistence.

#### 3.18.1. Root-Mean-Square Deviation (RMSD) Analysis

The structural stability was assessed by monitoring the RMSD of the protein backbone and the complexes (Figure 7C). For both AKT1 complexes, the RMSD values stabilized rapidly and remained low throughout the simulation: aromadendrene oxide-(2)-AKT1 (ligand: 0.025 nm; protein: 0.254 nm; complex: 0.256 nm) and isofuranodienone-AKT1 (ligand: 0.027 nm; protein: 0.260 nm; complex: 0.263 nm), indicating high stability. The FGFR1 complexes exhibited slightly higher fluctuations: aromadendrene oxide-(2)-FGFR1 (ligand: 0.027 nm; protein: 0.860 nm; complex: 0.859 nm) showed some random drift, while isofuranodienone-FGFR1 (ligand: 0.034 nm; protein: 0.564 nm; complex: 0.564 nm) stabilized after an initial 20 ns equilibration phase.

#### 3.18.2. Root-Mean-Square Fluctuation (RMSF) Analysis

Residue-level flexibility was evaluated by RMSF (Figure 7D). The average RMSF was lower for AKT1 complexes (aromadendrene oxide-(2)-AKT1: 0.136 nm; isofuranodienone-AKT1: 0.130 nm) compared to FGFR1 complexes (aromadendrene oxide-(2)-FGFR1: 0.449 nm; isofuranodienone-FGFR1: 0.206 nm), suggesting a more rigid binding interface, particularly for the AKT1 complexes.

#### 3.18.3. Hydrogen Bond Analysis

Intermolecular hydrogen bonds were analyzed to assess interaction stability (Figure 7E). The aromadendrene oxide-(2)-AKT1 and isofuranodienone-AKT1 complexes were each stabilized by one persistent hydrogen bond. For FGFR1, the aromadendrene oxide-(2)-FGFR1 complex maintained two hydrogen bonds, while isofuranodienone-FGFR1 was stabilized by one. The consistent presence of these bonds contributed significantly to complex stability.

#### 3.18.4. Solvent-Accessible Surface Area (SASA) Analysis

SASA was monitored to evaluate structural compactness (Figure 7F). All systems showed stable SASA profiles with no unfolding-related peaks. The average SASA values were: aromadendrene oxide-(2)-AKT1 (166.59 nm^2^), isofuranodienone-AKT1 (167.19 nm^2^), aromadendrene oxide-(2)-FGFR1 (306.95 nm^2^), and isofuranodienone-FGFR1 (303.15 nm^2^), indicating that ligand binding did not induce significant structural unfolding.

#### 3.18.5. Radius of Gyration (Rg) Analysis

Protein compactness was evaluated using Rg (Figure 7G). The average Rg values remained stable throughout the simulation: aromadendrene oxide-(2)-AKT1 and isofuranodienone-AKT1 (2.03 nm), aromadendrene oxide-(2)-FGFR1 (3.10 nm), and isofuranodienone-FGFR1 (2.82 nm), confirming that the proteins maintained their structural integrity without notable expansion or collapse.

## 4. Discussion

Chronic exposure to UVB radiation (280–320 nm) is a major environmental contributor to skin photodamage, initiating a pathological cascade characterized by oxidative stress [43], sustained inflammation, and an increased risk of photocarcinogenesis [44,45]. Despite the substantial health and aesthetic burden associated with photoaging, effective clinical interventions remain limited, highlighting the urgent need for novel therapeutic agents and advanced delivery strategies. *Codonopsis pilosula*, a traditional food-medicine homologous material, has been reported to possess anti-aging and health-promoting properties [46]. This traditional background provided the initial rationale for our study. However, the bioactivity of its lipophilic components, particularly in the context of skin photoaging, remains insufficiently explored. To address this gap, the selection of the lipophilic fraction was further supported by ethnopharmacological evidence. Traditional records describe *Codonopsis pilosula* root as a remedy for “weakness and premature aging,” and its frequent inclusion in health-promoting dietary formulas suggests a long-standing, though empirically based, association with longevity and skin vitality. To date, most pharmacological validation has concentrated on hydrophilic macromolecules, whereas the lipophilic pool—rich in volatile terpenoids and fatty acid derivatives and more amenable to transdermal delivery—has been overlooked. Thus, our study translates the traditional anti-aging indication of *Codonopsis pilosula* into a scientifically testable hypothesis by targeting its lipophilic constituents, and further validates this choice through GC-MS identification of known bioactive markers (e.g., paeonol and ethyl linoleate) that possess documented antioxidant and anti-inflammatory activities. Nevertheless, key unresolved questions include their precise molecular targets and strategies to overcome inherent physicochemical limitations such as poor skin permeability and stability for effective topical delivery.

To address the delivery challenge, we developed an integrated topical platform (CP-L nanogel). This system was designed to concurrently solve the penetration and retention dilemmas, establishing a robust platform for delivering challenging bioactive compounds like CP-L. The design exploits the deformability of transferosomes, achieved through the incorporation of NaDC as an edge activator to enhance membrane flexibility, a defining feature that distinguishes TFs from conventional liposomes [47]. This structural property, supported by the elliptical morphology observed by TEM [48], is expected to facilitate improved skin permeation. To further overcome the limited cutaneous retention associated with liquid nanocarriers, CP-L-TFs were incorporated into a Pluronic-based thermosensitive hydrogel. The formulation exhibited a critical sol–gel transition at approximately 32 °C, allowing convenient application and rapid gelation upon contact with the skin, thereby ensuring localized delivery and minimizing runoff [49]. Physicochemical characterization confirmed the physical encapsulation of vesicles within the gel matrix. Incorporation of the vesicles altered the microstructure of the hydrogel, resulting in a rougher cross-section and reduced pore size [50], a structural modification expected to regulate diffusion and promote sustained drug release. Moreover, the CP-L-TFs exhibited good storage stability at 4 °C over 7 days, with no significant changes in particle size or encapsulation efficiency, supporting the practical feasibility of the formulation. Thus, the CP-L nanogel system synergizes the penetration-enhancing properties of deformable TFs with the superior retention of a thermosensitive hydrogel. Crucially, the superior delivery capability of this design was validated functionally: flow cytometry demonstrated significantly enhanced cellular internalization of a model fluorescent probe encapsulated in the nanogel compared to its free form. This quantitative evidence (1.8-fold increase in fluorescence intensity) is not merely a proof of concept but direct validation that the system overcomes the bioavailability bottleneck of free CP-L. By synergistically enhancing penetration (via deformable vesicles) and prolonging retention (via the thermosensitive gel), this integrated platform systematically optimizes drug exposure at the target site, providing the foundational rationale for the observed therapeutic superiority.

Using this optimized formulation, its therapeutic effects were evaluated in well-established UV-induced photoaging models both in vivo and in vitro. The CP-L nanogel achieved superior restoration across multiple phenotypic and molecular indicators compared to unformulated CP-L. In the mouse model, this was evidenced by the significant attenuation of UV-induced epidermal hyperplasia and marked improvement in skin texture [51]. These in vivo findings were robustly corroborated at the cellular level: in UVB-irradiated HaCaT keratinocytes, the CP-L nanogel outperformed the unformulated extract by significantly increasing cell viability and reducing apoptosis. Collectively, these data confirmed that the nanogel system not only preserves but substantially enhances the therapeutic efficacy of CP-L. In addition, the CP-L nanogel showed favorable hemocompatibility, with a hemolysis rate below 3% at concentrations up to 40 μg/mL, indicating low risk of acute irritation upon topical application.

The enhanced efficacy is linked to the mitigation of core pathological drivers. UV radiation damages the dermal extracellular matrix by inducing ROS generation, triggering oxidative stress, and upregulating MMPs [52,53,54]. Consistent with these mechanisms, CP-L nanogel markedly increased the activities of key antioxidant enzymes (SOD, CAT, GSH-Px) and reduced MDA levels in skin tissue. Parallel in vitro assays showed the nanogel more potently scavenged intracellular ROS and restored antioxidant enzyme activities in HaCaT cells compared to free CP-L. Furthermore, photoaging involves persistent inflammation [55]. CP-L nanogel significantly downregulated key pro-inflammatory cytokines (TNF-α, IL-1α, IL-1β, IL-6) and MMPs in photoaged mouse skin [56,57], an effect consistently observed in HaCaT cells where it suppressed IL-6, TNF-α, and MMP-1 while promoting type I collagen synthesis. The simultaneous attenuation of oxidative stress, inflammation, and extracellular matrix degradation not only validates the multi-targeted mode of action of CP-L nanogel but also highlights its potential to disrupt the self-perpetuating cycle of damage that characterizes chronic photoaging. This coordinated multi-target effect aligns well with the recently proposed ‘oxidative stress–inflammation–ECM remodeling axis’ as a core therapeutic framework for combating UVB-induced skin photoaging [58]. Together, these coordinated effects underscore the capacity of the nanogel to comprehensively counteract the key molecular pathways driving UV-induced skin deterioration.

To elucidate the upstream molecular mechanism, transcriptomic analysis identified the PI3K–AKT signaling axis as a central mediator of the therapeutic response. This finding directed attention to its upstream regulator, FGFR, given its established role in activating PI3K–AKT signaling and its involvement in skin homeostasis [30,59]. Subsequent experimental validation across multiple levels solidified this insight. In vivo and in vitro, CP-L nanogel treatment effectively suppressed the UV-induced overexpression and phosphorylation of key components along the FGFR–PI3K–AKT–mTOR–p70S6K cascade. Notably, a recent study on a traditional Chinese herbal oil formulation demonstrated that plant-derived lipophilic extracts can exert therapeutic effects in inflammatory skin conditions through modulation of mTOR-related signaling [60], supporting the mechanistic plausibility of our findings regarding mTOR pathway inhibition by CP-L nanogel. Most conclusively, the functional dependence of CP-L nanogel’s efficacy on this pathway was demonstrated using the pharmacological inhibitor PD173074. The complete abrogation of its cytoprotective and signaling-inhibitory effects upon FGFR blockade provides direct causal evidence that targeting this axis is central to its mode of action. These results delineated a clear signaling cascade for CP-L nanogel operation and provide a mechanistic basis for its observed multi-target efficacy, functionally linking the modulation of this specific pathway to the coordinated amelioration of oxidative stress, inflammation, and ECM degradation.

To bridge the observed pathway inhibition with specific bioactive constituents, computational studies were employed. Molecular docking predicted that two major lipophilic constituents of CP-L—isofuranodienone and aromadendrene oxide-(2)—could bind with high affinity to FGFR1 and AKT1. These predictions were substantiated by molecular dynamics simulations, which showed that the resulting complexes remained stable over 100 ns, with favorable binding free energies and persistent interactions. These computational insights provide a plausible structural basis for the observed pathway inhibition. This successful integration of computational prediction and experimental validation not only identifies promising candidate molecules within CP-L but also strengthens the mechanistic narrative by directly linking specific phytochemical structures to the modulation of a key signaling axis in photoaging.

In summary, this study demonstrated that the CP-L nanogel alleviated skin photoaging through a coordinated mechanism involving enhanced topical delivery, potent antioxidant and anti-inflammatory actions, and specific inhibition of the FGFR/PI3K-AKT-mTOR pathway. The proposed mechanism of action is comprehensive and well-supported: (1) the nanogel system enhanced the topical delivery and cellular uptake of active constituents; (2) it specifically inhibited UV-induced activation of the FGFR/PI3K-AKT-mTOR-p70S6K signaling cascade; (3) via modulation of this axis, it concurrently alleviated oxidative stress and inflammatory responses; and (4) ultimately, it promoted collagen biosynthesis and restores dermal architecture. These interconnected effects have been consistently validated across both in vivo and in vitro models. Collectively, this study is the first to develop an integrated topical delivery platform for CP-L and to elucidate that its anti-photoaging efficacy is mechanistically grounded in the suppression of the FGFR/PI3K/AKT/mTOR axis. These findings position the CP-L nanogel as a mechanistically defined, multi-targeted topical strategy with promising potential for preventing and treating skin photoaging. Its origin from a food-medicine homologous material also lays a solid foundation for developing safe and effective novel skincare products or topical formulations. Certainly, this study has several limitations that also suggest productive avenues for future investigation. First, CP-L is a complex mixture, and while isofuranodienone and aromadendrene oxide-(2) were identified as key bioactive candidates by computational simulations, their independent biological activity requires experimental validation in future studies. Second, the in vivo dose was selected based on preliminary experiments, but a full dose–response evaluation was not performed; systematic dose-dependent studies are needed to establish the optimal regimen and safety margin. Third, our mechanistic validation relied primarily on a single pharmacological inhibitor (PD173074). Although supported by multiple complementary lines of evidence—transcriptomics, RT-qPCR, Western blot, and computational analyses—additional genetic approaches (e.g., siRNA silencing or pathway activators) would provide more definitive causal evidence. Fourth, while in vitro hemolysis and cytotoxicity assays support a favorable preliminary safety profile, long-term in vivo safety data—including dermal irritation, sensitization, and chronic toxicity—remain essential for clinical translation and will be prioritized in future work. Fifth, the findings are derived from HaCaT cells and mouse models, which may not fully recapitulate human skin complexity; studies using three-dimensional skin equivalents or human explants would strengthen translational potential. These acknowledged limitations do not undermine the validity of the conclusions drawn here but rather delineate the scope for continued exploration in this promising field.

## 5. Conclusions

In conclusion, this study demonstrated that a thermosensitive nanogel loaded with the lipophilic extract of *Codonopsis pilosula* effectively mitigates skin photoaging. The formulation exhibited superior efficacy over unformulated CP-L in both murine and cellular models by alleviating oxidative stress, suppressing inflammation and apoptosis, and promoting collagen synthesis—an effect attributed to the advanced delivery system that significantly enhances cellular uptake. These benefits were mediated through the specific inhibition of the FGFR/PI3K-AKT-mTOR-p70S6K signaling pathway, as validated by transcriptomic analysis, RT-qPCR, Western blot, molecular docking, dynamics simulations, and functional blockade with an FGFR inhibitor. Collectively, these preclinical findings position the CP-L nanogel as a promising, mechanistically defined topical strategy derived from an edible plant source for the prevention and treatment of skin photoaging. Nevertheless, several limitations—including the lack of systematic dose–response evaluation in vivo, reliance on a single pharmacological inhibitor for pathway validation, and the absence of long-term safety data—warrant further investigation before clinical translation. Future studies addressing these aspects will be essential to consolidate the translational potential of this formulation.

## Figures and Tables

**Table 1:** Primers used for RT-qPCR.

Gene	Primer Sequence
mTOR	Forward: 5′- CAAACCACAGGGTGAGGACT-3′
	Reverse: 5′- AGGGCAGCAACAGTGAGAGT-3′
FGFR1	Forward: 5′- GGGTTGTATTTTACCCAGTAGGAGT-3′
	Reverse: 5′- AGGTAGGAAATGTCCTTATGTGCTT-3′
PI3K	Forward: 5′- TATTGCGAGGGAAGCGAGAC-3′
	Reverse: 5′- ACTTCGCCGTCTACCACTAC-3′
p70S6K	Forward: 5′- CGTGGAGTCTGCGGCG-3′
	Reverse: 5′- CATATGGTCCAACTCCCCCA-3′
AKT1	Forward: 5′- AAGAAGGAGGTCATCGTCGC-3′
	Reverse: 5′- CTTGAGGGCCGTAAGGAAGG-3′

**Table 2:** Chemical constituents identified in CP-L by GC-MS total ion chromatogram.

No.	Name	Retention Time (min)	Formula	Average RI	Relative Content (%)
1	Tetradecanoic acid	26.268	C_14_H_28_O_2_	1841.66	6.29%
2	Tridecanoic acid	36.004	C_13_H_26_O_2_	2303.31	4.20%
3	Linoleic acid ethyl ester	22.591	C_20_H_36_O_2_	1694.97	3.78%
4	Ethyl Oleate	7.541	C_20_H_38_O_2_	1091.18	3.65%
5	9,12-Octadecadienoic acid (Z, Z)-, methyl ester	25.213	C_19_H_34_O_2_	1799.48	3.55%
6	Isofuranodienone	35.632	C_15_H_18_O_2_	2277.8	3.50%
7	9,12-Octadecadienoic acid, methyl ester	16.11	C_19_H_34_O_2_	1441.24	2.81%
8	Coumarin	31.455	C_9_H_6_O_2_	2056.94	2.40%
9	Cedren-13-ol, 8-	5.29	C_15_H_24_O	978.64	2.11%
10	Caryophylla-4(12),8(13)-dien-5a-ol	2.865	C_15_H_24_O	791.86	2.08%
11	Aromadendrene oxide-(2)	16.455	C_15_H_24_O	1454.75	1.80%
12	9-Decenoic acid	16.729	C_10_H_18_O_2_	1464.86	1.70%
13	Ethanone, 1-(2-hydroxy-4-methoxyphenyl)-	35.322	C_9_H_10_O_3_	2259.09	1.68%
14	n-Decanoic acid	16.48	C_10_H_20_O_2_	1455.01	1.62%
15	Epicurzerenone	25.479	C_15_H_18_O_2_	1810.55	1.58%
16	cis-9-Hexadecenal	25.461	C_16_H_30_O	1808.42	1.54%
17	Hexanoic acid, 2-phenylethyl ester	3.069	C_14_H_20_O2	816.06	1.41%
18	Benzeneacetic acid, hexyl ester	26.334	C_14_H_20_O_2_	1844.87	1.36%
19	2-Propenoic acid, 3-phenyl-, ethyl ester	3.346	C_11_H_12_O_2_	843.63	1.34%
20	Pentadecanoic acid, methyl ester	29.819	C_16_H_32_O_2_	1986.96	1.33%

**Table 3:** Scoring criteria for dorsal skin condition in mice.

Score	Wrinkled Erythema	Hyperpigmentation	Scab
4	No red spots or wrinkles	No pigmentation	No scabbing
3	Slight red spots and fine wrinkles	A small amount of pigmentation	Slight scabbing
2	Moderate redness and wrinkles	Moderate pigmentation	Moderate scabbing
1	A large number of red spots and wrinkles	A large amount of pigmentation	A large number of scabs

## Data Availability

The original contributions presented in this study are included in the article. Further inquiries can be directed to the corresponding author(s).

## Abbreviations

AKT	Protein kinase B
CAT	Catalase
C6	Coumarin 6
CP-L	*Codonopsis pilosula* lipophilic extract
DEGs	Differentially expressed genes
ECM	Extracellular matrix
EVG	Elastin Van Gieson
FGFR	Fibroblast growth factor receptor
FT-IR	Fourier-transform infrared spectroscopy
GC-MS	Gas chromatography–mass spectrometry
GSH-Px	Glutathione peroxidase
HaCaT	Human immortalized keratinocyte cell line
H&E	Hematoxylin and eosin
IHC	Immunohistochemistry
IL-6	Interleukin-6
KEGG	Kyoto Encyclopedia of Genes and Genomes
MDA	Malondialdehyde
MED	Minimal erythema dose
MMP	Matrix metalloproteinase
MMP-1	Matrix metalloproteinase-1
mTOR	Mammalian target of rapamycin
NaDC	Sodium deoxycholate
PDI	Polydispersity index
PI3K	Phosphoinositide 3-kinase
ROS	Reactive oxygen species
RT-qPCR	Quantitative reverse transcription polymerase chain reaction
SOD	Superoxide dismutase
SPF	Specific pathogen-free
TEM	Transmission electron microscopy
TFs	Transferosomes
TNF-α	Tumor necrosis factor-alpha
UV	Ultraviolet
VE	Vitamin E
